# Identifying and prioritizing evidence needs in self-care interventions for sexual and reproductive health

**DOI:** 10.3389/fgwh.2023.1148244

**Published:** 2023-06-08

**Authors:** Gilda Sedgh, Annik Sorhaindo

**Affiliations:** ^1^ Independent Researcher, Philadelphia, PA, United States; ^2^ Independent Researcher, Mexico City, Mexico

**Keywords:** research agenda, self-managed abortion, HIV self-testing, self-injectable contraception, SRHR, self-care (MeSH)

## Abstract

**Background:**

Self-care as an extension of health care systems can increase access to care. The development of programs and generation of evidence to support self-care in sexual and reproductive health (SRH) is a relatively nascent field. We undertook a study to identify and prioritize evidence gaps for SRH self-care.

**Methods:**

We used the CHNRI methodology and administered two online surveys to stakeholders affiliated with major self-care networks. The first survey was used to identify evidence gaps, and the second to prioritize them using predetermined criteria.

**Results:**

We received 51 responses to the first survey and 36 responses to the second. Many evidence gaps focused on awareness of and demand for self-care options and best mechanisms for supporting users of self-care with information, counseling and linkages to care.

**Conclusion:**

A priority area of work ahead should be determining which aspects of the learning agenda reflect gaps in evidence and which reflect a need to effectively synthesize and disseminate existing evidence.

## Introduction

Self-care has the potential to transform healthcare by increasing access to information, services and support and reducing dependency on facility-based services, especially for people who currently face barriers to healthcare access. It can provide individuals with greater awareness, autonomy, and control over their own health and wellbeing. When integrated into a health system, self-care has the potential to benefit the system as a whole (1) and contribute to efforts to achieve universal health coverage (UHC) (2).

The World Health Organization (WHO) defines self-care as “the ability of individuals, families and communities to promote health, prevent disease, maintain health and cope with illness and disability with or without the support of a health care provider” (3). The WHO defines self-care interventions as tools that support self-care, including evidence-based, high-quality medications, devices, diagnostics and/or digital interventions that can be provided fully or partially outside formal health services and be used with or without a health worker.

Self-care interventions are well-suited for the delivery of sexual and reproductive health (SRH) care because individuals, especially those in low- and middle-income countries (LMICs), may lack access to affordable, accessible care or may avoid using facility-based services for fear of being stigmatized (3, 4). Self-care interventions in this space include, but are not limited to HIV self-testing, self-injection of subcutaneous injectable contraception (DMPA-SC), self-managed medical abortion, and antenatal self-care in accordance with WHO guidelines (5–7). Self-care is not a new phenomenon, but the development of codified national and global SRH self-care guidelines and programs to support self-care as part of the health care system is still a relatively nascent field. In 2019, the WHO published its first guideline providing evidence-based recommendations for key self-care interventions in sexual and reproductive health and rights (SRHR) (3); the guidelines were most recently updated in 2022, and have been expanded to address a broader range of self-care interventions in 2022 (7).

A critical component of efforts to advance quality self-care as part of health systems is a clear understanding of the evidence that stakeholders—including policymakers, program implementers, advocates, and funders—need to advance their objectives (8). The WHO guideline included considerations for future research identified during the process of developing and updating the self-care guidelines (9). That effort focused on evidence needed to inform future guidelines and produced a set of illustrative (rather than priority) research questions. A key feature of the WHO self-care guideline development process was an intentional effort to incorporate a gender, equity, and human rights lens in the shaping of the guideline recommendations and the research agenda (9).

To our knowledge, the field has not yet generated a global self-care learning agenda that is relevant across SRH interventions and that focuses on policies and program design and implementation of self-care. The U.S. Agency for International Development -funded Research for Scalable Solutions project supported six countries to develop family planning research and learning agendas whose aims included to expand the development, adoption, and implementation of family planning approaches, at scale (10); these included but were not focused on self-care. Two scoping reviews focused on evidence related to self-managed medical abortion used systematic approaches to locate and inventory existing research and highlight gaps in available evidence, but these reviews did not include a process for identifying evidence needed by stakeholders outside the research community (11, 12). To address the gap for a stakeholder-informed learning agenda for a range of SRH self-care interventions and guide future investments in knowledge generation in the field, we undertook a systematic process to identify and prioritize evidence gaps and develop a learning agenda for SRH self-care.

It has been argued that both researchers and practitioners should be engaged in research agenda-setting processes; practitioners generate recommendations that are useful for decision-making, and researchers focus on whether questions can be answered with a well-designed study (13). A recently published framework for research utilization similarly argues the importance of employing a collaborative agenda-setting process (13). For these reasons, to develop a learning agenda in SHR self-care, we employed an adaptation of the Child Health and Nutrition Research Initiative (CHNRI). The CHNRI is a systematic and transparent priority–setting process that engages a range of stakeholders in the process of establishing and prioritizing evidence gaps (14). It has been used for setting research priorities for global child health, mental health, diabetes, adolescent health and health policy and education, among other topics (15).

We set out to identify and prioritize evidence gaps and establish a learning agenda for a range of SRH self-care interventions, through online consultation with professionals engaged in SRH self-care from different perspectives. The primary audience for this learning agenda is national and international policymakers, researchers and monitoring and evaluation specialists, program managers and civil society organizations responsible for promoting self-care interventions, and donors investing in relevant knowledge generation.

## Methods

The CHNRI approach involves following specific steps to identify high priority evidence gaps. First, to generate a bank of learning questions that represent gaps in the SRH self-care evidence base on a range of perspectives, we administered a survey to stakeholders engaged in SRH self-care, asking respondents to identify evidence needs (see Supplementary Figure S1). The survey asked respondents to identify needs that are relevant to all SRH self-care, and, separately, evidence needs that are specific to each of four major self-care interventions: self-injectable contraception, HIV self-testing (HIVST), self-managed abortion, and self-management of antenatal care (ANC). We focused on these four interventions because they seem to have gained the most traction so far among the range of self-care interventions in SRHR. We did not provide formal definitions of self-care with respect to each of these interventions in the surveys. We circulated this survey by email in March 2022 using the Google forms platform to networks of professionals in self-care in SRH in global spaces, namely the Self-Care Trailblazers Group (SCTG) (16) (which had a membership of roughly 260 at the time of the survey), WHO's “Implementing Best Practices” (IBP) points of contact (17), and the Reproductive Health Supplies Coalition's caucus on new and emerging technologies (18). The exact numbers of recipients of this survey is not known because we were not apprised of the sizes of the latter two listservs and we expect that some people were members of more than one of these groups. In addition, we asked individuals receiving the emails to forward the survey to other professionals who may be appropriate respondents. Our interest with this sampling approach was to bring forth and gather information from experts rather than a representative sample of members of these groups and listservs. We deleted duplicate evidence gaps and responses that we could not decipher, framed the evidence gaps as learning questions, and organized the learning questions into domains that arose from the questions themselves.

To guide the prioritization of the evidence gaps identified in the first survey, we first established criteria for assessing these evidence gaps. We developed a list of five potential criteria, drawing from prior applications of the CHNRI (19). To minimize the burden on survey respondents, we used three that were most relevant to this process. These criteria were impact, feasibility, and answerability, defined as follows:

• **Impact**: Would filling this evidence gap provide knowledge that is useful to stakeholders?
• **Feasibility:** Can the evidence gap be filled with a reasonable budget and in a reasonable amount of time (<2 years)?
• **Answerability:** Is the evidence gap well-defined and is the product or endpoint well-framed?

The two criteria that we did not use were generalizability (whether the findings would be relevant to other populations) and equity (whether filling the knowledge gap would reduce inequities in access to care). We administered a second survey in May 2022, in which we asked respondents to rate the learning questions generated in the first survey against the criteria named above (see Supplementary Figure S2). We sought a more limited group of respondents to the second survey, with a focus on those who would likely have sufficient appreciation for research methods to be able to assess the feasibility of the learning questions and their answerability, as well as an appreciation for the potential impact of answering the learning questions. Unlike the first survey, a diversity of viewpoints was not essential for the second survey. For these reasons, we circulated this survey only to members of the SCTG's Evidence and Learning Working Group. Some individuals might have responded to just one survey, and some might have responded to both.

For each of the learning questions identified in the first survey, respondents were asked to indicate whether answering the learning question met each of the criteria above. Respondents had the options of answering yes, no, or not sure, and we converted these responses to 1.0, 0 and 0.5, respectively. For each learning question and criterion, we computed the average of all respondents' scores. We then summed the average scores across the three criteria to get a total score for each learning question. This approach gave equal weight to each criterion. In the instructions accompanying both surveys, we asked that respondents only respond to questions on topic areas in which they feel they have expertise.

We received guidance at key stages of this work from the Evidence Mapping and Prioritization Sub working Group (SWG). The SWG is comprised of seventeen members of the ELWG with relevant expertise who volunteered to provide technical guidance to this workstream. We piloted both surveys with the SWG before administering them more widely. The SWG members also weighed in on the final selection of assessment criteria above, and recommended stakeholders to whom we could administer the survey.

As with prior applications of the CHNRI methodology (20, 21), and in accordance with the institutional review board (IRB) guidelines of Population Services International (22), IRB approval was not deemed necessary for this study because the interview questions focused on the respondents' professional knowledge of a topic, rather than information about their personal experiences. Potential respondents were not pressured to participate in the survey.

## Results

We received 51 responses to the first survey, in which we asked respondents to identify evidence gaps. Most (60%) of respondents reported their primary affiliation as being a non-governmental organization, 21% worked in service delivery, and the remainder represented donor agencies, academia, governments and multilateral agencies (Table 1). More than half (55%) of respondents reported having at least 12 years of experience in their field and another 20% said they had 8–12 years of experience. Most respondents also said they have moderate or high levels of expertise in each of the SRH self-care interventions (Supplementary Figure S3). The majority 61%) of respondents indicated that their work is focused on Africa, and18% indicated their work has a global or multi-regional focus. The developing region represented by the fewest respondents was Asia (4%). After cleaning the responses and merging duplicates, we identified 17–36 unique learning questions for each self-care intervention.

**Table 1:** Survey respondents’ years of professional experience and region of focus.

	Survey #1	Survey #2
	*n* = 51	*n* = 36
	%	%
**Years of experience**		
1–3 years	6	3
4–7 years	14	11
8–12 years	20	17
>12 years	55	69
Not given	6	0
**Region of focus**		
Africa	61	50
Asia	4	11
LAC	16	3
Europe	2	0
Multiple or global	18	36
**Primary affiliation**		
NGO	60	–
Service delivery	21	–
Donor	6	–
Academic	4	–
Other*	8	–
	100.0	100.0

^*^ Includes multilateral agency, government, and drug manufacturing.

We received 36 responses to the second survey, in which we asked respondents to assess learning questions. Of these, 50% (18) indicated that they work primarily in sub-Saharan Africa, 11% focused on Asia, and 36% indicated that their work had a global or multi-regional focus. We classified each of the ten learning questions that earned the highest scores for each intervention in one the following four domains: (1) enabling policy and regulatory environment; (2) knowledge, attitudes and preference related to self-care; (3) support for users of self-care, including linkages to follow-up care; and (4) equitable access to care. A few questions that did not fit into any of these domains are grouped separately (Table 2). All of the learning questions, their average scores on each criterion and their composite scores can be found in Supplementary Tables S1–S5.

**Table 2:** Self-care learning questions by intervention and domain.

Enabling policy and regulatory environment	Knowledge, attitudes and preferences related to self-care	Service delivery and support for users of self-care	Reducing inequities in access to care, including self-care	Other
**HIV self-testing (HIVST)**				
	What are people's reasons for seeking HIVST?	How can we make HIVST easy to use and accessible to users?	Which populations have the greatest need for HIVST?	What is the impact of HIVST on the number of people diagnosed with HIV?
	How can we drive interest in using quality-assured HIVST products?	Do users of HIVST need pre-test support?	What sub-groups are not reached with HIVST?	
		How is pre-test counselling for HIVST best implemented?		
		How can we encourage users to report HIVST results?		
		What are best ways to support clients who receive a positive result?		
		How can privacy be ensured through the HIVST process?		
**Self-injectable DMPA-SC**				
Which countries have policies that support and/or promote self-injection?	What proportion of women know about self-injectable contraception?	What are effective and efficient approaches to supporting provider-client SI counseling, training, and support?		How do clients store DMPA-SC at home?
	When presented the choice between provider and self-injection, how do user preferences vary by their characteristics?	What are provider perceptions about training clients to self-inject?		
	How supportive are male partners of SI users?			
	Are users of the self-injectable willing to refer others to the same method?			
	What are the barriers to self-injection from the point of view of women?			
	What are women's reasons for discontinuing self-injection?			
**Self-managed abortion**				
In which countries is self-managed abortion legally allowed?		How can telemedicine be effectively used to support self-managed abortion in the global south?	Which groups of women should self-managed abortion services be targeted to?	
		How can information to support self-managed abortion be delivered through digital means in legally restricted contexts?	What are promising and/or effective models for supporting access to self-managed abortion in humanitarian, fragile and legally restrictive settings?	
		How do the most effective approaches to providing women with information on sources of drugs and support for self-managed abortion vary by setting?		
		What mechanisms can be used to make post-abortion contraceptive services available to women who self-manage their abortions?		
		What are effective strategies for linking self-managed abortion with facility-based services, for women who need facility-based follow-up care?		
		How can we increase supportive treatment of abortion clients when women who self-manage abortions require facility-based care?		
		Are health service personnel sensitized to provide supportive, comprehensive services to vulnerable populations who use self-managed abortion?		
**Self-managed antenatal care**				
	What are effective ways to improve community knowledge about antenatal self-care?	What environment is required to support self-care in the antenatal period?		Which self-care behaviors during pregnancy are most critical to promote in order to optimize maternal and newborn health?
		How can we best empower women with information on why and how to use self-care interventions in the antenatal period?		Is antenatal self-care cost-effective?
		What approaches to antenatal self-care are being effectively used, and how can we learn from them?		What are the limits of antenatal self-care?
		How can women be trained to identify danger signs in pregnancy?		How do we standardize automatic blood pressure checking machines for blood pressure checks at home?
		How effective is maternal education for self-care?		
**SRHR self-care—general**				
What are most effective approaches to promoting self-care with policy makers?	Why and under what circumstances do people choose self-care for SRHR instead of care from a provider?	Can digital solutions be used to monitor quality of self-care?	What are the barriers and opportunities for advancing SRHR self-care in humanitarian and fragile settings?	Does self-care result in cost savings to the health care system?
What financially sustainable models can be developed to make self-care products available in procurement systems of countries?	What is the level of people's knowledge about self-care?	What can be done to make the promotion of self-care options more appealing to health care workers?		What are the key indicators for measuring self-care?
				Are there cost savings to users of self-care?

### Learning questions

#### Enabling policy and regulatory environment

In the category of SRH broadly, there was an interest in understanding effective approaches to promoting self-care with policy makers, and financially sustainable models for making self-care products available in procurement systems. For both self-injectable contraception and self-managed abortion, there was a perceived need to understand which countries have policies in place supporting self-care. None of the ten highest priority evidence gaps on HIV self-testing or ANC self-care pertained to the policy or regulatory enabling environment.

#### Knowledge, attitudes and preferences

With respect to SRH self-care in general, stakeholders cited a need for evidence on why and under what circumstances people choose self-care for SRH. Respondents noted a need for evidence on levels of awareness of self-care methods and how to raise awareness (at both the individual and the community levels), and levels of demand for self-care, together with factors that drive demand. These areas of learning arose mostly with reference to self-injectable DMPA-SC but also with reference to HIV self-testing. One of the learning questions on antenatal care also fell in this category.

#### Support for users of self-care

With respect to learning questions that cut across SRH interventions, two key questions pertained to how to make self-care options more appealing to health care workers and how to use digital solutions to monitor and ensure the quality of self-care. More than half of the intervention-specific priority learning questions were directed at how to support clients using self-care with information and counseling before, during and after the process of using self-care tools. This was especially an area of focus with respect to HIVST, self-managed abortion and ANC. Learning questions related to HIVST addressed the need for evidence on pre-test support and counseling; ensuring privacy during the self-testing process; and encouraging users to report their results. For self-managed abortion, questions focused on how to support women with information to help them manage their abortions, including in legally restrictive contexts; effective strategies for linking women to facility-based care as needed; and how to ensure facility-based care providers provide quality, stigma free service.

#### Equitable access to self-care/reaching neglected populations

For SRH self-care in general, respondents also pointed to a need to understand the barriers and opportunities for advancing self-care in humanitarian and fragile settings. Some of the learning questions on self-managed abortion and HIVST pertained to how we can expand the reach of health services with these methods. For HIVST, questions focused on identifying populations with the greatest need and the groups that have not yet been reached. Questions on self-managed abortion additionally asked how programs can support women in humanitarian, fragile and legally restrictive settings.

## Discussion

To the best of our knowledge, the SRH self-care learning agenda presented here is the first of its kind, in that the learning questions were named and prioritized by a large number of stakeholders who use evidence in their work.

The domains reflected the themes that emerged from the learning questions that stakeholders named. More than half of the priority learning questions that were intervention-specific were directed at how to support clients using self-care with information and counseling before, during and after the process of using self-care. Many also focused on the need for evidence on awareness of and demand for self-care interventions. One of the promises of self-care is the potential to reach previously underserved populations, such as people living in fragile or humanitarian settings, and stakeholders also expressed a need for evidence on how to effectively reach these populations.

The WHO also explored research gaps in the process of developing SRH self-care guidelines—but the published guidelines included a set of *illustrative* research questions, and these were not intended to be a comprehensive list of topics that merit further research (4). Moreover, the WHO did not undertake a prioritization process to identify the evidence gaps that are most critical to fill. Nevertheless, it is notable that there are some common themes across the WHO's research agendas and the priority learning questions identified through this CHNRI. For example, both the WHO and this process highlighted a lack of evidence pertaining to user preferences, acceptability of self-care options and equitable access to care.

Different classifications of the learning questions could potentially help researchers and other stakeholders to develop a responsive research agenda. For example, priority areas suggested by our findings include: (1) evaluation of self-care interventions for the general population and for vulnerable populations; (2) studies of the cost-effectiveness of self-care interventions; (3) identification of barriers and facilitators to self-care interventions; (4) the testing of new tools and resources to support users of self-care interventions, and (5) the actual and potential population impacts of self-care interventions.

Our approach was subject to a few limitations. First, our findings were derived from a non-random convenience of respondents and we cannot be sure that the respondents had a clear line of sight into evidence needed to support self-care policies and programs. However, the fact that most had many years of experience in SRH and were affiliated with the professional groups to which we circulated the surveys, and that they approached their work from a range of perspectives as reflected in their organizational affiliations, suggests that the respondents were an appropriate target group for these surveys on the whole. Another limitation is that the CHNRI process did not allow for a group discussion and idea generation process in which people's inputs can build on those of their colleagues. Finally, while our respondents represented a broad range of geographies, we were not able to identify country-specific or region-specific evidence gaps, even though research priorities might be different for different countries or regions.

We were also unable to determine which of the learning questions that arose from this process represent gaps in evidence or gaps in stakeholder knowledge—that is, for some of the learning questions, it is possible that a body of evidence exists but respondents were not familiar with the evidence, for example because it had not been effectively disseminated. We conducted rapid scans of the literature on the five highest priority evidence gaps in each intervention and we did not find a large body of evidence related to them. It is nevertheless possible that the evidence exists in gray literature or that more systematic literature reviews would uncover more information relevant to some of the learning questions. Such reviews were beyond the scope of this work.

One of the potential criteria for assessing the learning questions was whether answering the question would help reduce inequities in access to health care and in health outcomes. This criterion was not ultimately used to assess the learning questions, but research aimed at answering many of the questions can and should apply an equity lens.

Going forward, one priority area of work that should be undertaken is a process for determining which of the learning questions reflect gaps in evidence or gaps in stakeholder knowledge, which reflects a need to more effectively disseminate existing evidence. This could entail scoping reviews and systematic reviews of existing literature, followed by syntheses and dissemination of existing evidence to relevant audiences where this a body of evidence already.

This learning agenda was generated through a process that was systematic, transparent, collaborative and replicable. It allowed us to reach a range of stakeholders engaged in work across low– and middle–income countries in a short time period, and ensured that various groups engaged in the agenda setting process. We hope that the learning agenda presented here will contribute to efficient investments in research that can advance policies and programs supporting the implementation of self-care.

## Data Availability

The raw data supporting the conclusions of this article will be made available by the authors, without undue reservation.
