# Supplementary material for: Identifying and prioritizing evidence needs in self-care interventions for sexual and reproductive health
Source: Front Glob Womens Health. 2023 Jun 8;4:1148244. doi: 10.3389/fgwh.2023.1148244 (PMC10285388; doi:10.3389/fgwh.2023.1148244)
Supplement: Supplementary file 1 [file Datasheet1.zip › 1. Sedgh and Sorhaindo_Supplementary Figure 1.pdf]

Identifying evidence needs for self-care interventions in sexual and reproductive health and rights (SRHR)

BACKGROUND: The Evidence and Learning Working Group (ELWG) has recently launched an Evidence Mapping and Prioritization Workstream in order to identify and prioritize evidence needs in self-care interventions for sexual and reproductive health and rights (SRHR).

DEFINITION OF SELF CARE: Self-care interventions are high-quality drugs, devices, diagnostics and/or digital interventions that can be provided fully or partially outside formal health services and be used with or without a health worker. Self-care in SRHR includes, but is not limited to HIV self-testing, self-injection of DMPA-SC, self-managed abortion and self-care during antenatal care and delivery.

PURPOSE OF THIS SURVEY: The purpose of this survey is to identify learning questions – including evidence gaps and research questions -- pertaining to self-care in SRHR which, if answered, would help make evidence-based self-care interventions in SRHR widely available in low resource settings.

INSTRUCTIONS: When suggesting learning questions in SRHR self-care, please think about those that can be filled by well-designed studies with clear endpoints, and whose findings will be useful to stakeholders engaged in SRHR self-care policy, program planning, advocacy, research and funding. FOR EXAMPLE: “Does self-care reduce the cost of care to the end user?”

At a later stage, we will rank these questions, giving priority to those that (a) are high impact (that is, filling the gap would provide knowledge that would be most useful to stakeholders), (b) can feasibly be addressed with a reasonable budget in in a reasonable amount of time) and (c) will help reduce inequities in access to safe, high-quality care.

There are only ~20 questions and you are welcome to skip the questions on SRHR interventions that you are not familiar with or not professionally engaged with.

NOTE: We will only report your responses in combination with responses from other people.

Thank you in advance for your support of this work.

* Required

Self-managed abortion

INSTRUCTIONS: Please think about learning questions that can be answered with a well-designed study with clear endpoints, and whose findings will be useful to stakeholders in SRHR self-care.

REMINDER: Feel free to skip this section if you are not familiar with self-managed abortion.

In your view, what are the most important learning questions pertaining to SELF-MANAGED ABORTION? Please name up to three (3) questions.

1. Self-managed abortion: learning question 1

____

2. Self-managed abortion: learning question 2

____

3. Self-managed abortion: learning question 3

____

4. What would you say is your level of expertise in self-managed abortion?

   Mark only one oval.

   - ⬭ High
   - ⬭ Moderate
   - ⬭ Little or none

DMPA-SC

INSTRUCTIONS: Please think about learning questions that can be answered with a well-designed study with clear endpoints, and whose findings will be useful to stakeholders in SRHR self-care.

REMINDER: Feel free to skip this section if you are not familiar with DMPA-SC.

Question 3: In your view, what are the most important learning questions pertaining to SELF-INJECTABLE DMPA-SC? Please name up to three (3) questions.

5. DMPA-SC: learning question 1

6. DMPA-SC: learning question 2

7. DMPA-SC: learning question 3

8. What would you say is your level of expertise in self-injectable DMPA-SC?

Mark only one oval.

- High
- Moderate
- Little or none

HIV self-testing

INSTRUCTIONS: Please think about learning questions that can be answered with a well-designed study with clear endpoints, and whose findings will be useful to stakeholders in SRHR self-care.

REMINDER: Feel free to skip this section if you are not familiar with HIV self-testing.

Question 4: In your view, what are the most important learning questions pertaining to HIV SELF-TESTING? Please name up to three (3) questions.

9. HIV self-testing: learning question 1

____

10. HIV self-testing: learning question 2

____

11. HIV self-testing: learning question 3

____

12. What would you say is your level of expertise in HIV testing?

Mark only one oval.

- ( ) High
- ( ) Moderate
- ( ) Little or none

Self-care in the antenatal period

INSTRUCTIONS: Please think about learning questions that can be answered with a well-designed study with clear endpoints, and whose findings will be useful to stakeholders in SRHR self-care.

REMINDER: Feel free to skip this section if you are not familiar with antenatal period.

Question 5: In your view, what are the most important learning questions pertaining to SELF-CARE IN THE ANTENATAL PERIOD? Please name up to three (3) questions.

13. Self-care in the antenatal period: learning question 1

____

14. Self-care in the antenatal period: learning question 2

____

15. Self-care in the antenatal period: learning question 3

____

16. What would you say is your level of expertise in self-care in the antenatal period?

Mark only one oval.

- ◯ High
- ◯ Moderate
- ◯ Little or none

SRHR self-care general

INSTRUCTIONS:

When naming learning questions in SRHR self-care, please think about those that can be filled with a well-designed study with clear endpoints, and whose findings will be useful to stakeholders in SRHR self-care.

In your view, what are the most important learning questions in SRHR self-care BROADLY? Please name up to three (3) questions.

17. Broad SRHR self-care: learning question 1

____

18. Broad SRHR self-care: learning question 2

____

19. Broad SRHR self-care: learning question 3

____

About you

20. What is your primary affiliation? *

Check all that apply.

- [ ] Multilateral/UN agency
- [ ] Government agency or ministry
- [ ] Non-governmental organization (NGO)
- [ ] Donor agency or foundation
- [ ] Academic institution
- [ ] Hospital, health clinic, or other health providing organization
- [ ] Manufacturer, pharmaceutical company or laboratory
- [ ] Consultant / Independent
- [ ] Other: _______________________________

21. If your affiliation falls into more than one category, what is the second category?

Check all that apply.

- [ ] Multilateral/UN agency
- [ ] Government agency or ministry
- [ ] Non-governmental agency (NGO)
- [ ] Donor agency or foundation
- [ ] Academic institution
- [ ] Hospital, health clinic, or other health providing organization
- [ ] Manufacturer, pharmaceutical company or laboratory
- [ ] Consultant / Independent
- [ ] Other: ____________________

22. How many years have you worked in your general field? *

Mark only one oval.

- ( ) 1-3
- ( ) 4-7
- ( ) 8-12
- ( ) More than 12

23. Which region does your work primarily focus on?

Check all that apply.

- [ ] Asia
- [ ] Africa
- [ ] Europe
- [ ] Latin America and the Caribbean
- [ ] North America
- [ ] Global

24. If you work in a specific country or countries, please specify here.

Check all that apply.

- [ ] Afghanistan
- [ ] Albania
- [ ] Algeria
- [ ] Andorra
- [ ] Angola
- [ ] Antigua and Barbuda
- [ ] Argentina
- [ ] Armenia
- [ ] Australia
- [ ] Austria
- [ ] Azerbaijan
- [ ] Bahamas
- [ ] Bahrain
- [ ] Bangladesh
- [ ] Barbados
- [ ] Belarus
- [ ] Belgium
- [ ] Belize
- [ ] Benin
- [ ] Bhutan
- [ ] Bolivia (Plurinational State of)
- [ ] Bosnia and Herzegovina
- [ ] Botswana
- [ ] Brazil
- [ ] Brunei Darussalam
- [ ] Bulgaria
- [ ] Burkina Faso
- [ ] Burundi
- [ ] Cabo Verde
- [ ] Cambodia
- [ ] Cameroon
- [ ] Canada
- [ ] Central African Republic
- [ ] Chad
- [ ] Chile
- [ ] China
- [ ] Colombia

- [ ] Comoros
- [ ] Congo
- [ ] Cook Islands
- [ ] Costa Rica
- [ ] Côte d'Ivoire
- [ ] Croatia
- [ ] Cuba
- [ ] Cyprus
- [ ] Czechia
- [ ] Democratic People's Republic of Korea
- [ ] Democratic Republic of the Congo
- [ ] Denmark
- [ ] Djibouti
- [ ] Dominica
- [ ] Dominican Republic
- [ ] Ecuador
- [ ] Egypt
- [ ] El Salvador
- [ ] North Macedonia
- [ ] Eritrea
- [ ] Estonia
- [ ] Eswatini
- [ ] Ethiopia
- [ ] Fiji
- [ ] Finland
- [ ] France
- [ ] Gabon
- [ ] Gambia
- [ ] Georgia
- [ ] Germany
- [ ] Ghana
- [ ] Greece
- [ ] Grenada
- [ ] Guatemala
- [ ] Guinea
- [ ] Seychelles
- [ ] Guyana
- [ ] Haiti
- [ ] Honduras
- [ ] Hungary

- [ ] Iceland
- [ ] India
- [ ] Indonesia
- [ ] Iran (Islamic Republic of)
- [ ] Iraq
- [ ] Ireland
- [ ] Israel
- [ ] Italy
- [ ] Jamaica
- [ ] Japan
- [ ] Jordan
- [ ] Kazakhstan
- [ ] Kenya
- [ ] Kiribati
- [ ] Kuwait
- [ ] Kyrgyzstan
- [ ] Lao People's Democratic Republic
- [ ] Latvia
- [ ] Lebanon
- [ ] Lesotho
- [ ] Liberia
- [ ] Libya
- [ ] Lithuania
- [ ] Luxembourg
- [ ] Madagascar
- [ ] Malawi
- [ ] Malaysia
- [ ] Maldives
- [ ] Mali
- [ ] Malta
- [ ] Marshall Islands
- [ ] Mauritania
- [ ] Mauritius
- [ ] Mexico
- [ ] Micronesia (Federated States of)
- [ ] Monaco
- [ ] Mongolia
- [ ] Montenegro
- [ ] Morocco
- [ ] Mozambique

- [ ] Myanmar
- [ ] Namibia
- [ ] Nauru
- [ ] Nepal
- [ ] Netherlands
- [ ] New Zealand
- [ ] Nicaragua
- [ ] Niger
- [ ] Nigeria
- [ ] Niue
- [ ] Norway
- [ ] Oman
- [ ] Pakistan
- [ ] Palau
- [ ] Panama
- [ ] Papua New Guinea
- [ ] Paraguay
- [ ] Peru
- [ ] Philippines
- [ ] Poland
- [ ] Portugal
- [ ] Qatar
- [ ] Republic of Korea
- [ ] Republic of Moldova
- [ ] Romania
- [ ] Russian Federation
- [ ] Rwanda
- [ ] Saint Kitts and Nevis
- [ ] Saint Lucia
- [ ] Saint Vincent and the Grenadines
- [ ] Samoa
- [ ] San Marino
- [ ] Sao Tome and Principe
- [ ] Saudi Arabia
- [ ] Senegal
- [ ] Serbia
- [ ] Sierra Leone
- [ ] Singapore
- [ ] Slovakia
- [ ] Slovenia

- [ ] Solomon Islands
- [ ] Somalia
- [ ] South Africa
- [ ] South Sudan
- [ ] Spain
- [ ] Sri Lanka
- [ ] Sudan
- [ ] Suriname
- [ ] Sweden
- [ ] Switzerland
- [ ] Syrian Arab Republic
- [ ] Tajikistan
- [ ] Thailand
- [ ] Timor-Leste
- [ ] Togo
- [ ] Tonga
- [ ] Trinidad and Tobago
- [ ] Tunisia
- [ ] Turkey
- [ ] Turkmenistan
- [ ] Tuvalu
- [ ] Uganda
- [ ] Ukraine
- [ ] United Arab Emirates
- [ ] United Kingdom of Great Britain and Northern Ireland
- [ ] United Republic of Tanzania
- [ ] United States of America
- [ ] Uruguay
- [ ] Uzbekistan
- [ ] Vanuatu
- [ ] Venezuela (Bolivarian Republic of)
- [ ] Viet Nam
- [ ] Yemen
- [ ] Zambia
- [ ] Zimbabwe

25. Any additional comments

26. Would you be willing to be contacted if we have follow-up questions?

Mark only one oval.

- Yes
- No
- Maybe

Contact information (optional)

27. Name

28. Email address

Thank you and next steps	We expect the findings to inform future investments in knowledge generation. THANK YOU FOR YOUR CONTRIBUTIONS TO THIS IMPORTANT ENDEAVOR.

This content is neither created nor endorsed by Google.

Google Forms
