# Supplementary material for: Identifying and prioritizing evidence needs in self-care interventions for sexual and reproductive health
Source: Front Glob Womens Health. 2023 Jun 8;4:1148244. doi: 10.3389/fgwh.2023.1148244 (PMC10285388; doi:10.3389/fgwh.2023.1148244)
Supplement: Supplementary file 1 [file Datasheet1.zip › 2. Sedgh and Sorhaindo_Supplementary Figure 2.pdf]

Assessing learning questions for self-care interventions in sexual and reproductive health and rights (SRHR)

BACKGROUND: The Evidence and Learning Working Group (ELWG) of the Self-Care Trailblazers' Group (SCTG) is seeking to identify and prioritize evidence needs to support self-care interventions for sexual and reproductive health and rights (SRHR) in low resource settings. Earlier in 2022, we circulated a survey asking stakeholders to name the evidence gaps that they face in their work.

PURPOSE OF THIS SURVEY: The purpose of this survey is to assess evidence gaps that the stakeholders identified. We will use your responses to these questions to identify the highest priority evidence gaps.

INSTRUCTIONS: For each learning question, please answer whether you think that filling answering the learning question would be (a) impactful and (b) feasible, and whether the learning question is (c) answerable; each of these is defined as follows:

IMPACTFUL: Filling the evidence gap would provide knowledge that would be very useful to stakeholders.

FEASIBLE: The evidence gap can be addressed with a reasonable budget and amount of time (<2 years).

ANSWERABLE: The evidence gap is well-defined and the product or endpoint is well-framed.

There are five intervention areas. Please select the interventions that you are familiar with. The questions for each intervention area should take 15-20 minutes to complete. You can take a break and come back without losing your work.

We will only report your responses in combination with responses from other people.

Thank you in advance for your support of this work.

* Required

1. This survey includes learning questions for five topic areas. Please choose the topic area you would like to assess. There will be an opportunity to assess other topic areas once you have completed your first choice. *

Mark only one oval.

- Self-managed medical abortion *Skip to question 2*
- HIV self-testing *Skip to question 30*
- DMPA-SC *Skip to question 59*
- Self-care in the antenatal period *Skip to question 94*
- SRHR self-care general *Skip to question 112*

Self-managed medical abortion	Self-managed abortion is when the person induces their own abortion with drugs recommended by the WHO outside of a health care setting.

2. SMA-1. What are effective approaches to providing women with information on sources of drugs and support for self-managed abortion, and how do best approaches vary by setting?

Mark only one oval per row.

	Yes	No	Don't know
**IMPACTFUL: Would filling this evidence gap provide knowledge that is useful to stakeholders?**	⬭	⬭	⬭
**FEASIBLE: Can the evidence gap be filled with a reasonable budget and amount of time?**	⬭	⬭	⬭
**ANSWERABLE: Is the evidence gap well-defined and is the product or endpoint well-framed?**	⬭	⬭	⬭

3. SMA-2. Are current strategies for supporting self-managed abortions successful in ensuring women's confidentiality?

Mark only one oval per row.

	Yes	No	Don't know
**IMPACTFUL: Would filling this evidence gap provide knowledge that is useful to stakeholders?**	◯	◯	◯
**FEASIBLE: Can the evidence gap be filled with a reasonable budget and amount of time?**	◯	◯	◯
**ANSWERABLE: Is the evidence gap well-defined and is the product or endpoint well-framed?**	◯	◯	◯

4. SMA-3. Are there - or should there be - health status requirements for eligibility for self-managed abortion?

Mark only one oval per row.

	Yes	No	Don't know
**IMPACTFUL: Would filling this evidence gap provide knowledge that is useful to stakeholders?**	⬭	⬭	⬭
**FEASIBLE: Can the evidence gap be filled with a reasonable budget and amount of time?**	⬭	⬭	⬭
**ANSWERABLE: Is the evidence gap well-defined and is the product or endpoint well-framed?**	⬭	⬭	⬭

5. SMA-4. What are effective strategies for linking self-managed abortion with facility-based services, for women who need facility-based follow-up care?

Mark only one oval per row.

	Yes	No	Don't know
**IMPACTFUL: Would filling this evidence gap provide knowledge that is useful to stakeholders?**			
**FEASIBLE: Can the evidence gap be filled with a reasonable budget and amount of time?**			
**ANSWERABLE: Is the evidence gap well-defined and is the product or endpoint well-framed?**			

6. SMA-5. How do we increase supportive treatment of abortion clients when women who self-manage abortions require facility-based care?

Mark only one oval per row.

	Yes	No	Don't know
**IMPACTFUL: Would filling this evidence gap provide knowledge that is useful to stakeholders?**	◯	◯	◯
**FEASIBLE: Can the evidence gap be filled with a reasonable budget and amount of time?**	◯	◯	◯
**ANSWERABLE: Is the evidence gap well-defined and is the product or endpoint well-framed?**	◯	◯	◯

7. SMA-6. Are health service personnel sensitized to provide supportive, comprehensive services to vulnerable populations who use SMA?

Mark only one oval per row.

	Yes	No	Don't know
**IMPACTFUL: Would filling this evidence gap provide knowledge that is useful to stakeholders?**	⬭	⬭	⬭
**FEASIBLE: Can the evidence gap be filled with a reasonable budget and amount of time?**	⬭	⬭	⬭
**ANSWERABLE: Is the evidence gap well-defined and is the product or endpoint well-framed?**	⬭	⬭	⬭

8. SMA-7. How can telemedicine be effectively used to support self-managed abortion in the global south?

Mark only one oval per row.

	Yes	No	Don't know
**IMPACTFUL: Would filling this evidence gap provide knowledge that is useful to stakeholders?**	⬭	⬭	⬭
**FEASIBLE: Can the evidence gap be filled with a reasonable budget and amount of time?**	⬭	⬭	⬭
**ANSWERABLE: Is the evidence gap well-defined and is the product or endpoint well-framed?**	⬭	⬭	⬭

9. SMA-8. How can information to support self-managed be delivered through digital means in legally restricted contexts?

Mark only one oval per row.

	Yes	No	Don't know
**IMPACTFUL: Would filling this evidence gap provide knowledge that is useful to stakeholders?**	⬭	⬭	⬭
**FEASIBLE: Can the evidence gap be filled with a reasonable budget and amount of time?**	⬭	⬭	⬭
**ANSWERABLE: Is the evidence gap well-defined and is the product or endpoint well-framed?**	⬭	⬭	⬭

10. SMA-9. What mechanisms can be used to make post-abortion contraceptive services available to women who self-manage their abortions?

Mark only one oval per row.

	Yes	No	Don't know
**IMPACTFUL: Would filling this evidence gap provide knowledge that is useful to stakeholders?**	◯	◯	◯
**FEASIBLE: Can the evidence gap be filled with a reasonable budget and amount of time?**	◯	◯	◯
**ANSWERABLE: Is the evidence gap well-defined and is the product or endpoint well-framed?**	◯	◯	◯

11. SMA-10. What is the potential for pharmacies and drug stores to provide comprehensive abortion care for self-managed abortion?

Mark only one oval per row.

	Yes	No	Don't know
**IMPACTFUL: Would filling this evidence gap provide knowledge that is useful to stakeholders?**			
**FEASIBLE: Can the evidence gap be filled with a reasonable budget and amount of time?**			
**ANSWERABLE: Is the evidence gap well-defined and is the product or endpoint well-framed?**			

12. SMA-11. What are effective strategies for making medication abortion available over the counter in the Global South?

Mark only one oval per row.

	Yes	No	Don't know
**IMPACTFUL: Would filling this evidence gap provide knowledge that is useful to stakeholders?**			
**FEASIBLE: Can the evidence gap be filled with a reasonable budget and amount of time?**			
**ANSWERABLE: Is the evidence gap well-defined and is the product or endpoint well-framed?**			

13. SMA-12. What are women and girls' perspectives, preferences and experiences around self-managed abortion in humanitarian and fragile settings?

Mark only one oval per row.

	Yes	No	Don't know
**IMPACTFUL: Would filling this evidence gap provide knowledge that is useful to stakeholders?**	⬭	⬭	⬭
**FEASIBLE: Can the evidence gap be filled with a reasonable budget and amount of time?**	⬭	⬭	⬭
**ANSWERABLE: Is the evidence gap well-defined and is the product or endpoint well-framed?**	⬭	⬭	⬭

14. SMA-13. What are promising and/or effective models for supporting access to self-managed abortion in humanitarian, fragile and legally restrictive settings?

Mark only one oval per row.

	Yes	No	Don't know
**IMPACTFUL: Would filling this evidence gap provide knowledge that is useful to stakeholders?**			
**FEASIBLE: Can the evidence gap be filled with a reasonable budget and amount of time?**			
**ANSWERABLE: Is the evidence gap well-defined and is the product or endpoint well-framed?**			

15. SMA-14. Does self-managed abortion reduce the risk of being stigmatized in health care facilities in developing countries where abortion is legally restrictive?

Mark only one oval per row.

	Yes	No	Don't know
**IMPACTFUL: Would filling this evidence gap provide knowledge that is useful to stakeholders?**	⬭	⬭	⬭
**FEASIBLE: Can the evidence gap be filled with a reasonable budget and amount of time?**	⬭	⬭	⬭
**ANSWERABLE: Is the evidence gap well-defined and is the product or endpoint well-framed?**	⬭	⬭	⬭

16. SMA-15. How can we reduce stigma around self-managed abortion?

Mark only one oval per row.

	Yes	No	Don't know
**IMPACTFUL: Would filling this evidence gap provide knowledge that is useful to stakeholders?**	⬭	⬭	⬭
**FEASIBLE: Can the evidence gap be filled with a reasonable budget and amount of time?**	⬭	⬭	⬭
**ANSWERABLE: Is the evidence gap well-defined and is the product or endpoint well-framed?**	⬭	⬭	⬭

17. SMA-16. What can we appropriately remunerate providers who support self-managed abortion?

Mark only one oval per row.

	Yes	No	Don't know
**IMPACTFUL: Would filling this evidence gap provide knowledge that is useful to stakeholders?**	⬭	⬭	⬭
**FEASIBLE: Can the evidence gap be filled with a reasonable budget and amount of time?**	⬭	⬭	⬭
**ANSWERABLE: Is the evidence gap well-defined and is the product or endpoint well-framed?**	⬭	⬭	⬭

18. SMA-17. Can self-managed abortion help reduce work loads for medical professionals in public or private settings?

Mark only one oval per row.

	Yes	No	Don't know
**IMPACTFUL: Would filling this evidence gap provide knowledge that is useful to stakeholders?**	⬭	⬭	⬭
**FEASIBLE: Can the evidence gap be filled with a reasonable budget and amount of time?**	⬭	⬭	⬭
**ANSWERABLE: Is the evidence gap well-defined and is the product or endpoint well-framed?**	⬭	⬭	⬭

19. SMA-18. In which countries is self-managed abortion legally allowed?

Mark only one oval per row.

	Yes	No	Don't know
**IMPACTFUL: Would filling this evidence gap provide knowledge that is useful to stakeholders?**	⬭	⬭	⬭
**FEASIBLE: Can the evidence gap be filled with a reasonable budget and amount of time?**	⬭	⬭	⬭
**ANSWERABLE: Is the evidence gap well-defined and is the product or endpoint well-framed?**	⬭	⬭	⬭

20. SMA-19. How well is self-managed abortion provision reflected in policies and guidelines, at the national level and within professional associations?

Mark only one oval per row.

	Yes	No	Don't know
**IMPACTFUL: Would filling this evidence gap provide knowledge that is useful to stakeholders?**	⬭	⬭	⬭
**FEASIBLE: Can the evidence gap be filled with a reasonable budget and amount of time?**	⬭	⬭	⬭
**ANSWERABLE: Is the evidence gap well-defined and is the product or endpoint well-framed?**	⬭	⬭	⬭

21. SMA-20. How effectively are guidelines and policies for SMA disseminated to women and providers?

Mark only one oval per row.

	Yes	No	Don't know
**IMPACTFUL: Would filling this evidence gap provide knowledge that is useful to stakeholders?**	◯	◯	◯
**FEASIBLE: Can the evidence gap be filled with a reasonable budget and amount of time?**	◯	◯	◯
**ANSWERABLE: Is the evidence gap well-defined and is the product or endpoint well-framed?**	◯	◯	◯

22. SMA-21. What type of evidence and communication strategies will lead to full application of the WHO guideline for self-managed abortion?

Mark only one oval per row.

	Yes	No	Don't know
**IMPACTFUL: Would filling this evidence gap provide knowledge that is useful to stakeholders?**	⬭	⬭	⬭
**FEASIBLE: Can the evidence gap be filled with a reasonable budget and amount of time?**	⬭	⬭	⬭
**ANSWERABLE: Is the evidence gap well-defined and is the product or endpoint well-framed?**	⬭	⬭	⬭

23. SMA-22. What is the incidence of self-managed abortion in different population sub-groups (defined by age, education, wealth, and residence)?

Mark only one oval per row.

	Yes	No	Don't know
**IMPACTFUL: Would filling this evidence gap provide knowledge that is useful to stakeholders?**	⬭	⬭	⬭
**FEASIBLE: Can the evidence gap be filled with a reasonable budget and amount of time?**	⬭	⬭	⬭
**ANSWERABLE: Is the evidence gap well-defined and is the product or endpoint well-framed?**	⬭	⬭	⬭

24. SMA-23. Are pills for self-managed abortion seen as a last resort or is it preferred to medically-assisted abortion?

Mark only one oval per row.

	Yes	No	Don't know
**IMPACTFUL: Would filling this evidence gap provide knowledge that is useful to stakeholders?**			
**FEASIBLE: Can the evidence gap be filled with a reasonable budget and amount of time?**			
**ANSWERABLE: Is the evidence gap well-defined and is the product or endpoint well-framed?**			

25. SMA-24. What are the gaps in the need for abortion care that SMA cannot fill?

Mark only one oval per row.

	Yes	No	Don't know
**IMPACTFUL: Would filling this evidence gap provide knowledge that is useful to stakeholders?**	◯	◯	◯
**FEASIBLE: Can the evidence gap be filled with a reasonable budget and amount of time?**	◯	◯	◯
**ANSWERABLE: Is the evidence gap well-defined and is the product or endpoint well-framed?**	◯	◯	◯

26. SMA-25. Which groups of women should self-managed abortion services be targeted to?

Mark only one oval per row.

	Yes	No	Don't know
**IMPACTFUL: Would filling this evidence gap provide knowledge that is useful to stakeholders?**	⬭	⬭	⬭
**FEASIBLE: Can the evidence gap be filled with a reasonable budget and amount of time?**	⬭	⬭	⬭
**ANSWERABLE: Is the evidence gap well-defined and is the product or endpoint well-framed?**	⬭	⬭	⬭

27. SMA-26. What percent of people who obtain pills for self-managed abortion end up using them?

Mark only one oval per row.

	Yes	No	Don't know
**IMPACTFUL: Would filling this evidence gap provide knowledge that is useful to stakeholders?**			
**FEASIBLE: Can the evidence gap be filled with a reasonable budget and amount of time?**			
**ANSWERABLE: Is the evidence gap well-defined and is the product or endpoint well-framed?**			

28. SMA-27. What proportion of women who use SMA have a subsequent unintended pregnancy?

Mark only one oval per row.

	Yes	No	Don't know
**IMPACTFUL: Would filling this evidence gap provide knowledge that is useful to stakeholders?**	⬭	⬭	⬭
**FEASIBLE: Can the evidence gap be filled with a reasonable budget and amount of time?**	⬭	⬭	⬭
**ANSWERABLE: Is the evidence gap well-defined and is the product or endpoint well-framed?**	⬭	⬭	⬭

29. This survey includes learning questions for five topic areas. Thank you for assessing learning questions on self-managed medical abortion. If you would like to assess ANOTHER topic area, please choose one below. You can also end the survey at this point, if you so choose. *

Mark only one oval.

- ⬭ Self-managed medical abortion *Skip to question 2*
- ⬭ HIV self-testing *Skip to question 30*
- ⬭ DMPA-SC *Skip to question 59*
- ⬭ Self-care in the antenatal period *Skip to question 94*
- ⬭ SRHR self-care general *Skip to question 112*
- ⬭ END SURVEY *Skip to question 149*

HIV self-testing

30. HIVST-1. What are people's reasons for seeking HIVST?

Mark only one oval per row.

	Yes	No	Don't know
**IMPACTFUL: Would filling this evidence gap provide knowledge that is useful to stakeholders?**	◯	◯	◯
**FEASIBLE: Can the evidence gap be filled with a reasonable budget and amount of time?**	◯	◯	◯
**ANSWERABLE: Is the evidence gap well-defined and is the product or endpoint well-framed?**	◯	◯	◯

31. HIVST-2. Which populations have the greatest need for HIV self-testing?

Mark only one oval per row.

	Yes	No	Don't know
**IMPACTFUL: Would filling this evidence gap provide knowledge that is useful to stakeholders?**			
**FEASIBLE: Can the evidence gap be filled with a reasonable budget and amount of time?**			
**ANSWERABLE: Is the evidence gap well-defined and is the product or endpoint well-framed?**			

32. HIVST-3. What sub-groups are not reached with HIV self-testing?

Mark only one oval per row.

	Yes	No	Don't know
**IMPACTFUL: Would filling this evidence gap provide knowledge that is useful to stakeholders?**			
**FEASIBLE: Can the evidence gap be filled with a reasonable budget and amount of time?**			
**ANSWERABLE: Is the evidence gap well-defined and is the product or endpoint well-framed?**			

33. HIVST-4. How can we drive interest in using quality-assured HIVST products?

Mark only one oval per row.

	Yes	No	Don't know
**IMPACTFUL: Would filling this evidence gap provide knowledge that is useful to stakeholders?**	⬭	⬭	⬭
**FEASIBLE: Can the evidence gap be filled with a reasonable budget and amount of time?**	⬭	⬭	⬭
**ANSWERABLE: Is the evidence gap well-defined and is the product or endpoint well-framed?**	⬭	⬭	⬭

34. HIVST-5. How can we make HIV self-testing easy to use and accessible to users?

Mark only one oval per row.

	Yes	No	Don't know
**IMPACTFUL: Would filling this evidence gap provide knowledge that is useful to stakeholders?**	⬭	⬭	⬭
**FEASIBLE: Can the evidence gap be filled with a reasonable budget and amount of time?**	⬭	⬭	⬭
**ANSWERABLE: Is the evidence gap well-defined and is the product or endpoint well-framed?**	⬭	⬭	⬭

35. HIVST-6. Do users of HIVST need pre-test support?

Mark only one oval per row.

	Yes	No	Don't know
**IMPACTFUL: Would filling this evidence gap provide knowledge that is useful to stakeholders?**			
**FEASIBLE: Can the evidence gap be filled with a reasonable budget and amount of time?**			
**ANSWERABLE: Is the evidence gap well-defined and is the product or endpoint well-framed?**			

36. HIVST-7. How is pre-test counselling implemented for HIVST?

Mark only one oval per row.

	Yes	No	Don't know
**IMPACTFUL: Would filling this evidence gap provide knowledge that is useful to stakeholders?**	⬭	⬭	⬭
**FEASIBLE: Can the evidence gap be filled with a reasonable budget and amount of time?**	⬭	⬭	⬭
**ANSWERABLE: Is the evidence gap well-defined and is the product or endpoint well-framed?**	⬭	⬭	⬭

37. HIVST-8. How can privacy be ensured with HIV self-testing?

Mark only one oval per row.

	Yes	No	Don't know
**IMPACTFUL: Would filling this evidence gap provide knowledge that is useful to stakeholders?**	⬭	⬭	⬭
**FEASIBLE: Can the evidence gap be filled with a reasonable budget and amount of time?**	⬭	⬭	⬭
**ANSWERABLE: Is the evidence gap well-defined and is the product or endpoint well-framed?**	⬭	⬭	⬭

38. HIVST-9. How can we encourage users to report HIV self-test results?

Mark only one oval per row.

	Yes	No	Don't know
**IMPACTFUL: Would filling this evidence gap provide knowledge that is useful to stakeholders?**			
**FEASIBLE: Can the evidence gap be filled with a reasonable budget and amount of time?**			
**ANSWERABLE: Is the evidence gap well-defined and is the product or endpoint well-framed?**			

39. HIVST-10. What is the impact of HIVST on the number of people diagnosed with HIV?

Mark only one oval per row.

	Yes	No	Don't know
**IMPACTFUL: Would filling this evidence gap provide knowledge that is useful to stakeholders?**			
**FEASIBLE: Can the evidence gap be filled with a reasonable budget and amount of time?**			
**ANSWERABLE: Is the evidence gap well-defined and is the product or endpoint well-framed?**			

40. HIVST-11. What are best ways to support clients who receive a positive result?

Mark only one oval per row.

	Yes	No	Don't know
**IMPACTFUL: Would filling this evidence gap provide knowledge that is useful to stakeholders?**	⬭	⬭	⬭
**FEASIBLE: Can the evidence gap be filled with a reasonable budget and amount of time?**	⬭	⬭	⬭
**ANSWERABLE: Is the evidence gap well-defined and is the product or endpoint well-framed?**	⬭	⬭	⬭

41. HIVST-12. How common are incorrect results from self-testing?

Mark only one oval per row.

	Yes	No	Don't know
**IMPACTFUL: Would filling this evidence gap provide knowledge that is useful to stakeholders?**			
**FEASIBLE: Can the evidence gap be filled with a reasonable budget and amount of time?**			
**ANSWERABLE: Is the evidence gap well-defined and is the product or endpoint well-framed?**			

42. HIVST-13. What proportion of people who self-test use post-test services?

Mark only one oval per row.

	Yes	No	Don't know
**IMPACTFUL: Would filling this evidence gap provide knowledge that is useful to stakeholders?**	◯	◯	◯
**FEASIBLE: Can the evidence gap be filled with a reasonable budget and amount of time?**	◯	◯	◯
**ANSWERABLE: Is the evidence gap well-defined and is the product or endpoint well-framed?**	◯	◯	◯

43. HIVST-14. Where do those who test positive on a self-test end up seeking further care?

Mark only one oval per row.

	Yes	No	Don't know
**IMPACTFUL: Would filling this evidence gap provide knowledge that is useful to stakeholders?**	◯	◯	◯
**FEASIBLE: Can the evidence gap be filled with a reasonable budget and amount of time?**	◯	◯	◯
**ANSWERABLE: Is the evidence gap well-defined and is the product or endpoint well-framed?**	◯	◯	◯

44. HIVST-15. How can we strengthen follow-up linkages to HIV self-testing ?

Mark only one oval per row.

	Yes	No	Don't know
**IMPACTFUL: Would filling this evidence gap provide knowledge that is useful to stakeholders?**			
**FEASIBLE: Can the evidence gap be filled with a reasonable budget and amount of time?**			
**ANSWERABLE: Is the evidence gap well-defined and is the product or endpoint well-framed?**			

45. HIVST-16. What is the role of mHealth in strengthening linkage to HIV care following self-testing?

Mark only one oval per row.

	Yes	No	Don't know
**IMPACTFUL: Would filling this evidence gap provide knowledge that is useful to stakeholders?**	◯	◯	◯
**FEASIBLE: Can the evidence gap be filled with a reasonable budget and amount of time?**	◯	◯	◯
**ANSWERABLE: Is the evidence gap well-defined and is the product or endpoint well-framed?**	◯	◯	◯

46. HIVST-17. How can provider perceptions on HIVST be modified to encourage acceptability of HIVST results?

Mark only one oval per row.

	Yes	No	Don't know
**IMPACTFUL: Would filling this evidence gap provide knowledge that is useful to stakeholders?**			
**FEASIBLE: Can the evidence gap be filled with a reasonable budget and amount of time?**			
**ANSWERABLE: Is the evidence gap well-defined and is the product or endpoint well-framed?**			

47. HIVST-18. What is the effect of HIVST on treatment for HIV?

Mark only one oval per row.

	Yes	No	Don't know
**IMPACTFUL: Would filling this evidence gap provide knowledge that is useful to stakeholders?**			
**FEASIBLE: Can the evidence gap be filled with a reasonable budget and amount of time?**			
**ANSWERABLE: Is the evidence gap well-defined and is the product or endpoint well-framed?**			

48. HIVST-19. How can we actively screen for and manage intimate partner violence following self-testing?

Mark only one oval per row.

	Yes	No	Don't know
**IMPACTFUL: Would filling this evidence gap provide knowledge that is useful to stakeholders?**			
**FEASIBLE: Can the evidence gap be filled with a reasonable budget and amount of time?**			
**ANSWERABLE: Is the evidence gap well-defined and is the product or endpoint well-framed?**			

49. HIVST-20. Can HIVST be packaged with a pregnancy test for sexual violence survivors?

Mark only one oval per row.

	Yes	No	Don't know
**IMPACTFUL: Would filling this evidence gap provide knowledge that is useful to stakeholders?**	⬭	⬭	⬭
**FEASIBLE: Can the evidence gap be filled with a reasonable budget and amount of time?**	⬭	⬭	⬭
**ANSWERABLE: Is the evidence gap well-defined and is the product or endpoint well-framed?**	⬭	⬭	⬭

50. HIVST-21. How can we ensure that users of HIVST have adequate emotional support?

Mark only one oval per row.

	Yes	No	Don't know
**IMPACTFUL: Would filling this evidence gap provide knowledge that is useful to stakeholders?**			
**FEASIBLE: Can the evidence gap be filled with a reasonable budget and amount of time?**			
**ANSWERABLE: Is the evidence gap well-defined and is the product or endpoint well-framed?**			

51. HIVST-22. How do you teach clients how to properly store testing kits?

Mark only one oval per row.

	Yes	No	Don't know
**IMPACTFUL: Would filling this evidence gap provide knowledge that is useful to stakeholders?**			
**FEASIBLE: Can the evidence gap be filled with a reasonable budget and amount of time?**			
**ANSWERABLE: Is the evidence gap well-defined and is the product or endpoint well-framed?**			

52. HIVST-23. How are HIVST kits disposed of?

Mark only one oval per row.

	Yes	No	Don't know
**IMPACTFUL: Would filling this evidence gap provide knowledge that is useful to stakeholders?**	◯	◯	◯
**FEASIBLE: Can the evidence gap be filled with a reasonable budget and amount of time?**	◯	◯	◯
**ANSWERABLE: Is the evidence gap well-defined and is the product or endpoint well-framed?**	◯	◯	◯

53. HIVST-24. What are the best ways to monitor disposal of the HIV ST kits?

Mark only one oval per row.

	Yes	No	Don't know
**IMPACTFUL: Would filling this evidence gap provide knowledge that is useful to stakeholders?**	⬭	⬭	⬭
**FEASIBLE: Can the evidence gap be filled with a reasonable budget and amount of time?**	⬭	⬭	⬭
**ANSWERABLE: Is the evidence gap well-defined and is the product or endpoint well-framed?**	⬭	⬭	⬭

54. HIVST-25. What proportion are used for first test vs. follow-up test after testing at a facility?

Mark only one oval per row.

	Yes	No	Don't know
**IMPACTFUL: Would filling this evidence gap provide knowledge that is useful to stakeholders?**	◯	◯	◯
**FEASIBLE: Can the evidence gap be filled with a reasonable budget and amount of time?**	◯	◯	◯
**ANSWERABLE: Is the evidence gap well-defined and is the product or endpoint well-framed?**	◯	◯	◯

55. HIVST-26. How can we measure HIVST utilization at scale using routine data systems?

Mark only one oval per row.

	Yes	No	Don't know
**IMPACTFUL: Would filling this evidence gap provide knowledge that is useful to stakeholders?**			
**FEASIBLE: Can the evidence gap be filled with a reasonable budget and amount of time?**			
**ANSWERABLE: Is the evidence gap well-defined and is the product or endpoint well-framed?**			

56. HIVST-27. How can HIV self-testing be made cost-effective?

Mark only one oval per row.

	Yes	No	Don't know
**IMPACTFUL: Would filling this evidence gap provide knowledge that is useful to stakeholders?**			
**FEASIBLE: Can the evidence gap be filled with a reasonable budget and amount of time?**			
**ANSWERABLE: Is the evidence gap well-defined and is the product or endpoint well-framed?**			

57. HIVST-28. How can HIVST be integrated into other SRH programs to reduce stigmatisation?

Mark only one oval per row.

	Yes	No	Don't know
**IMPACTFUL: Would filling this evidence gap provide knowledge that is useful to stakeholders?**	⬭	⬭	⬭
**FEASIBLE: Can the evidence gap be filled with a reasonable budget and amount of time?**	⬭	⬭	⬭
**ANSWERABLE: Is the evidence gap well-defined and is the product or endpoint well-framed?**	⬭	⬭	⬭

58. This survey includes learning questions for five topic areas. Thank you for assessing learning questions on HIV self-testing. If you would like to assess ANOTHER topic area, please choose one below. You can also end the survey at this point, if you so choose. *

Mark only one oval.

- ⬭ Self-managed medical abortion *Skip to question 2*
- ⬭ HIV self-testing *Skip to question 30*
- ⬭ DMPA-SC *Skip to question 59*
- ⬭ Self-care in the antenatal period *Skip to question 94*
- ⬭ SRHR self-care general *Skip to question 112*
- ⬭ END SURVEY *Skip to question 149*

DMPA-SC

59. DMPA-SC 1. What proportion of women know about self-injectable contraception?

Mark only one oval per row.

	Yes	No	Don't know
**IMPACTFUL: Would filling this evidence gap provide knowledge that is useful to stakeholders?**	⬭	⬭	⬭
**FEASIBLE: Can the evidence gap be filled with a reasonable budget and amount of time?**	⬭	⬭	⬭
**ANSWERABLE: Is the evidence gap well-defined and is the product or endpoint well-framed?**	⬭	⬭	⬭

60. DMPA-SC 2. When presented the choice between provider and self-injection, which do users prefer? How does this vary by users' characteristics?

Mark only one oval per row.

	Yes	No	Don't know
**IMPACTFUL: Would filling this evidence gap provide knowledge that is useful to stakeholders?**	◯	◯	◯
**FEASIBLE: Can the evidence gap be filled with a reasonable budget and amount of time?**	◯	◯	◯
**ANSWERABLE: Is the evidence gap well-defined and is the product or endpoint well-framed?**	◯	◯	◯

61. DMPA-SC 3. Can self-assessment tools for determining eligibility for self-injection be improved?

Mark only one oval per row.

	Yes	No	Don't know
**IMPACTFUL: Would filling this evidence gap provide knowledge that is useful to stakeholders?**	◯	◯	◯
**FEASIBLE: Can the evidence gap be filled with a reasonable budget and amount of time?**	◯	◯	◯
**ANSWERABLE: Is the evidence gap well-defined and is the product or endpoint well-framed?**	◯	◯	◯

62. DMPA-SC 4. What is the best approach to empowering women and girls to safely self-inject?

Mark only one oval per row.

	Yes	No	Don't know
**IMPACTFUL: Would filling this evidence gap provide knowledge that is useful to stakeholders?**	⬭	⬭	⬭
**FEASIBLE: Can the evidence gap be filled with a reasonable budget and amount of time?**	⬭	⬭	⬭
**ANSWERABLE: Is the evidence gap well-defined and is the product or endpoint well-framed?**	⬭	⬭	⬭

63. DMPA-SC 5. What are effective and efficient approaches to supporting provider-client SI counseling, training, and support?

Mark only one oval per row.

	Yes	No	Don't know
**IMPACTFUL: Would filling this evidence gap provide knowledge that is useful to stakeholders?**	⬭	⬭	⬭
**FEASIBLE: Can the evidence gap be filled with a reasonable budget and amount of time?**	⬭	⬭	⬭
**ANSWERABLE: Is the evidence gap well-defined and is the product or endpoint well-framed?**	⬭	⬭	⬭

64. DMPA-SC 6. What are the levels of uptake and continuation of self-injectable DMPA-SC?

Mark only one oval per row.

	Yes	No	Don't know
**IMPACTFUL: Would filling this evidence gap provide knowledge that is useful to stakeholders?**	⬭	⬭	⬭
**FEASIBLE: Can the evidence gap be filled with a reasonable budget and amount of time?**	⬭	⬭	⬭
**ANSWERABLE: Is the evidence gap well-defined and is the product or endpoint well-framed?**	⬭	⬭	⬭

65. DMPA-SC 7. What factors are associated with uptake and continuation of DMPA-SC?

Mark only one oval per row.

	Yes	No	Don't know
**IMPACTFUL: Would filling this evidence gap provide knowledge that is useful to stakeholders?**	⬭	⬭	⬭
**FEASIBLE: Can the evidence gap be filled with a reasonable budget and amount of time?**	⬭	⬭	⬭
**ANSWERABLE: Is the evidence gap well-defined and is the product or endpoint well-framed?**	⬭	⬭	⬭

66. DMPA-SC 8a. What potential side effects should be communicated to clients?

Mark only one oval per row.

	Yes	No	Don't know
**IMPACTFUL: Would filling this evidence gap provide knowledge that is useful to stakeholders?**	◯	◯	◯
**FEASIBLE: Can the evidence gap be filled with a reasonable budget and amount of time?**	◯	◯	◯
**ANSWERABLE: Is the evidence gap well-defined and is the product or endpoint well-framed?**	◯	◯	◯

67. DMPA-SC 8b. How can side effects be managed?

Mark only one oval per row.

	Yes	No	Don't know
**IMPACTFUL: Would filling this evidence gap provide knowledge that is useful to stakeholders?**	⬭	⬭	⬭
**FEASIBLE: Can the evidence gap be filled with a reasonable budget and amount of time?**	⬭	⬭	⬭
**ANSWERABLE: Is the evidence gap well-defined and is the product or endpoint well-framed?**	⬭	⬭	⬭

68. DMPA-SC 9. What is the level of user satisfaction with DMPA-SC?

Mark only one oval per row.

	Yes	No	Don't know
**IMPACTFUL: Would filling this evidence gap provide knowledge that is useful to stakeholders?**			
**FEASIBLE: Can the evidence gap be filled with a reasonable budget and amount of time?**			
**ANSWERABLE: Is the evidence gap well-defined and is the product or endpoint well-framed?**			

69. DMPA-SC 10. What are the prevalence and consequences of incorrect use?

Mark only one oval per row.

	Yes	No	Don't know
**IMPACTFUL: Would filling this evidence gap provide knowledge that is useful to stakeholders?**	◯	◯	◯
**FEASIBLE: Can the evidence gap be filled with a reasonable budget and amount of time?**	◯	◯	◯
**ANSWERABLE: Is the evidence gap well-defined and is the product or endpoint well-framed?**	◯	◯	◯

70. DMPA-SC 11. What are the potential adverse events associated with self-injectable SC, and what is their prevalence?

Mark only one oval per row.

	Yes	No	Don't know
**IMPACTFUL: Would filling this evidence gap provide knowledge that is useful to stakeholders?**	⬭	⬭	⬭
**FEASIBLE: Can the evidence gap be filled with a reasonable budget and amount of time?**	⬭	⬭	⬭
**ANSWERABLE: Is the evidence gap well-defined and is the product or endpoint well-framed?**	⬭	⬭	⬭

71. DMPA-SC 12. What are women's reasons for discontinuing self-injection?

Mark only one oval per row.

	Yes	No	Don't know
**IMPACTFUL: Would filling this evidence gap provide knowledge that is useful to stakeholders?**			
**FEASIBLE: Can the evidence gap be filled with a reasonable budget and amount of time?**			
**ANSWERABLE: Is the evidence gap well-defined and is the product or endpoint well-framed?**			

72. DMPA-SC 13. What are the barriers to self-injection from the point of view of women?

Mark only one oval per row.

	Yes	No	Don't know
**IMPACTFUL: Would filling this evidence gap provide knowledge that is useful to stakeholders?**	⬭	⬭	⬭
**FEASIBLE: Can the evidence gap be filled with a reasonable budget and amount of time?**	⬭	⬭	⬭
**ANSWERABLE: Is the evidence gap well-defined and is the product or endpoint well-framed?**	⬭	⬭	⬭

73. DMPA-SC 14. What are provider perceptions about training clients to self-inject?

Mark only one oval per row.

	Yes	No	Don't know
**IMPACTFUL: Would filling this evidence gap provide knowledge that is useful to stakeholders?**	⬭	⬭	⬭
**FEASIBLE: Can the evidence gap be filled with a reasonable budget and amount of time?**	⬭	⬭	⬭
**ANSWERABLE: Is the evidence gap well-defined and is the product or endpoint well-framed?**	⬭	⬭	⬭

74. DMPA-SC 15. What is the level of community awareness of self-injectable DMPA-SC?

Mark only one oval per row.

	Yes	No	Don't know
**IMPACTFUL: Would filling this evidence gap provide knowledge that is useful to stakeholders?**	◯	◯	◯
**FEASIBLE: Can the evidence gap be filled with a reasonable budget and amount of time?**	◯	◯	◯
**ANSWERABLE: Is the evidence gap well-defined and is the product or endpoint well-framed?**	◯	◯	◯

75. DMPA-SC 16. What were the referral pathways of current SI users?

Mark only one oval per row.

	Yes	No	Don't know
**IMPACTFUL: Would filling this evidence gap provide knowledge that is useful to stakeholders?**	◯	◯	◯
**FEASIBLE: Can the evidence gap be filled with a reasonable budget and amount of time?**	◯	◯	◯
**ANSWERABLE: Is the evidence gap well-defined and is the product or endpoint well-framed?**	◯	◯	◯

76. DMPA-SC 17. Are users of the self-injectable willing to refer others to the same method?

Mark only one oval per row.

	Yes	No	Don't know
**IMPACTFUL: Would filling this evidence gap provide knowledge that is useful to stakeholders?**			
**FEASIBLE: Can the evidence gap be filled with a reasonable budget and amount of time?**			
**ANSWERABLE: Is the evidence gap well-defined and is the product or endpoint well-framed?**			

77. DMPA-SC 18. How supportive are male partners of SI users?

Mark only one oval per row.

	Yes	No	Don't know
**IMPACTFUL: Would filling this evidence gap provide knowledge that is useful to stakeholders?**	⬭	⬭	⬭
**FEASIBLE: Can the evidence gap be filled with a reasonable budget and amount of time?**	⬭	⬭	⬭
**ANSWERABLE: Is the evidence gap well-defined and is the product or endpoint well-framed?**	⬭	⬭	⬭

78. DMPA-SC 19. How do clients properly store DMPA-SC at home?

Mark only one oval per row.

	Yes	No	Don't know
**IMPACTFUL: Would filling this evidence gap provide knowledge that is useful to stakeholders?**	◯	◯	◯
**FEASIBLE: Can the evidence gap be filled with a reasonable budget and amount of time?**	◯	◯	◯
**ANSWERABLE: Is the evidence gap well-defined and is the product or endpoint well-framed?**	◯	◯	◯

79. DMPA-SC 20. Do users share their injectables with others?

Mark only one oval per row.

	Yes	No	Don't know
**IMPACTFUL: Would filling this evidence gap provide knowledge that is useful to stakeholders?**	⬭	⬭	⬭
**FEASIBLE: Can the evidence gap be filled with a reasonable budget and amount of time?**	⬭	⬭	⬭
**ANSWERABLE: Is the evidence gap well-defined and is the product or endpoint well-framed?**	⬭	⬭	⬭

80. DMPA-SC 21. How can we measure self-injection utilization at scale using routine data systems?

Mark only one oval per row.

	Yes	No	Don't know
**IMPACTFUL: Would filling this evidence gap provide knowledge that is useful to stakeholders?**			
**FEASIBLE: Can the evidence gap be filled with a reasonable budget and amount of time?**			
**ANSWERABLE: Is the evidence gap well-defined and is the product or endpoint well-framed?**			

81. DMPA-SC 22. What is the potential demand for SI from the private sector?

Mark only one oval per row.

	Yes	No	Don't know
**IMPACTFUL: Would filling this evidence gap provide knowledge that is useful to stakeholders?**			
**FEASIBLE: Can the evidence gap be filled with a reasonable budget and amount of time?**			
**ANSWERABLE: Is the evidence gap well-defined and is the product or endpoint well-framed?**			

82. DMPA-SC 23. How have drug shops and pharmacies impacted uptake, continued use and user experience of DMPA-SC?

Mark only one oval per row.

	Yes	No	Don't know
**IMPACTFUL: Would filling this evidence gap provide knowledge that is useful to stakeholders?**	⬭	⬭	⬭
**FEASIBLE: Can the evidence gap be filled with a reasonable budget and amount of time?**	⬭	⬭	⬭
**ANSWERABLE: Is the evidence gap well-defined and is the product or endpoint well-framed?**	⬭	⬭	⬭

83. DMPA-SC 24. What are barriers and facilitators to making self-injection available in private sector?

Mark only one oval per row.

	Yes	No	Don't know
**IMPACTFUL: Would filling this evidence gap provide knowledge that is useful to stakeholders?**	◯	◯	◯
**FEASIBLE: Can the evidence gap be filled with a reasonable budget and amount of time?**	◯	◯	◯
**ANSWERABLE: Is the evidence gap well-defined and is the product or endpoint well-framed?**	◯	◯	◯

84. DMPA-SC 25. What is the cost of SI product to the user?

Mark only one oval per row.

	Yes	No	Don't know
**IMPACTFUL: Would filling this evidence gap provide knowledge that is useful to stakeholders?**	⬭	⬭	⬭
**FEASIBLE: Can the evidence gap be filled with a reasonable budget and amount of time?**	⬭	⬭	⬭
**ANSWERABLE: Is the evidence gap well-defined and is the product or endpoint well-framed?**	⬭	⬭	⬭

85. DMPA-SC 26. Does SI confer cost savings at points of care, compared to other contraceptive methods?

Mark only one oval per row.

	Yes	No	Don't know
**IMPACTFUL: Would filling this evidence gap provide knowledge that is useful to stakeholders?**	⬭	⬭	⬭
**FEASIBLE: Can the evidence gap be filled with a reasonable budget and amount of time?**	⬭	⬭	⬭
**ANSWERABLE: Is the evidence gap well-defined and is the product or endpoint well-framed?**	⬭	⬭	⬭

86. DMPA-SC 27. Has uptake of self-injectable been slower than expected? If so, why?

Mark only one oval per row.

	Yes	No	Don't know
**IMPACTFUL: Would filling this evidence gap provide knowledge that is useful to stakeholders?**	◯	◯	◯
**FEASIBLE: Can the evidence gap be filled with a reasonable budget and amount of time?**	◯	◯	◯
**ANSWERABLE: Is the evidence gap well-defined and is the product or endpoint well-framed?**	◯	◯	◯

87. DMPA-SC 28. How can the global supply chain for the product be strengthened?

Mark only one oval per row.

	Yes	No	Don't know
**IMPACTFUL: Would filling this evidence gap provide knowledge that is useful to stakeholders?**	⬭	⬭	⬭
**FEASIBLE: Can the evidence gap be filled with a reasonable budget and amount of time?**	⬭	⬭	⬭
**ANSWERABLE: Is the evidence gap well-defined and is the product or endpoint well-framed?**	⬭	⬭	⬭

88. DMPA-SC 29. Which countries have policies that support and/or promote self-injection?

Mark only one oval per row.

	Yes	No	Don't know
**IMPACTFUL: Would filling this evidence gap provide knowledge that is useful to stakeholders?**	⬭	⬭	⬭
**FEASIBLE: Can the evidence gap be filled with a reasonable budget and amount of time?**	⬭	⬭	⬭
**ANSWERABLE: Is the evidence gap well-defined and is the product or endpoint well-framed?**	⬭	⬭	⬭

89. DMPA-SC 30. What is the feasibility and acceptability of introducing DMPA-SC into a humanitarian or fragile context?

Mark only one oval per row.

	Yes	No	Don't know
**IMPACTFUL: Would filling this evidence gap provide knowledge that is useful to stakeholders?**			
**FEASIBLE: Can the evidence gap be filled with a reasonable budget and amount of time?**			
**ANSWERABLE: Is the evidence gap well-defined and is the product or endpoint well-framed?**			

90. DMPA-SC 31. What are the most effective models for supporting self-injectable DMPA-SC in humanitarian or fragile settings?

Mark only one oval per row.

	Yes	No	Don't know
**IMPACTFUL: Would filling this evidence gap provide knowledge that is useful to stakeholders?**	⬭	⬭	⬭
**FEASIBLE: Can the evidence gap be filled with a reasonable budget and amount of time?**	⬭	⬭	⬭
**ANSWERABLE: Is the evidence gap well-defined and is the product or endpoint well-framed?**	⬭	⬭	⬭

91. DMPA-SC 32. Are there economic benefits to making self-injectable DMPA-SC available to vulnerable populations?

Mark only one oval per row.

	Yes	No	Don't know
**IMPACTFUL: Would filling this evidence gap provide knowledge that is useful to stakeholders?**	◯	◯	◯
**FEASIBLE: Can the evidence gap be filled with a reasonable budget and amount of time?**	◯	◯	◯
**ANSWERABLE: Is the evidence gap well-defined and is the product or endpoint well-framed?**	◯	◯	◯

92. DMPA-SC 33. How can we prepare as a field for a longer-acting DMPA-SC formulation?

Mark only one oval per row.

	Yes	No	Don't know
**IMPACTFUL: Would filling this evidence gap provide knowledge that is useful to stakeholders?**			
**FEASIBLE: Can the evidence gap be filled with a reasonable budget and amount of time?**			
**ANSWERABLE: Is the evidence gap well-defined and is the product or endpoint well-framed?**			

93. This survey includes learning questions for five topic areas. Thank you for assessing learning questions on DMPA-SC. If you would like to assess ANOTHER topic area, please choose one below. You can also end the survey at this point, if you so choose. *

Mark only one oval.

- Self-managed medical abortion *Skip to question 2*
- HIV self-testing *Skip to question 30*
- DMPA-SC *Skip to question 59*
- Self-care in the antenatal period *Skip to question 94*
- SRHR self-care general *Skip to question 112*
- END SURVEY *Skip to question 149*

Self-care in the antenatal period

94. SC-ANC 1. What is the state of knowledge about and attitudes toward antenatal self-care?

Mark only one oval per row.

	Yes	No	Don't know
**IMPACTFUL: Would filling this evidence gap provide knowledge that is useful to stakeholders?**	◯	◯	◯
**FEASIBLE: Can the evidence gap be filled with a reasonable budget and amount of time?**	◯	◯	◯
**ANSWERABLE: Is the evidence gap well-defined and is the product or endpoint well-framed?**	◯	◯	◯

95. SC-ANC 2. What are health care providers' views on antenatal self-care?

Mark only one oval per row.

	Yes	No	Don't know
**IMPACTFUL: Would filling this evidence gap provide knowledge that is useful to stakeholders?**			
**FEASIBLE: Can the evidence gap be filled with a reasonable budget and amount of time?**			
**ANSWERABLE: Is the evidence gap well-defined and is the product or endpoint well-framed?3**			

96. SC-ANC 3. What environment is required to support self-care in the antenatal period?

Mark only one oval per row.

	Yes	No	Don't know
**IMPACTFUL: Would filling this evidence gap provide knowledge that is useful to stakeholders?**			
**FEASIBLE: Can the evidence gap be filled with a reasonable budget and amount of time?**			
**ANSWERABLE: Is the evidence gap well-defined and is the product or endpoint well-framed?3**			

97. SC-ANC 4. How can we best empower women with information on why and how to use self-care interventions in the antenatal period?

Mark only one oval per row.

	Yes	No	Don't know
**IMPACTFUL: Would filling this evidence gap provide knowledge that is useful to stakeholders?**	⬭	⬭	⬭
**FEASIBLE: Can the evidence gap be filled with a reasonable budget and amount of time?**	⬭	⬭	⬭
**ANSWERABLE: Is the evidence gap well-defined and is the product or endpoint well-framed?3**	⬭	⬭	⬭

98. SC-ANC 5. How can media be used to promote antenatal self-care?

Mark only one oval per row.

	Yes	No	Don't know
**IMPACTFUL: Would filling this evidence gap provide knowledge that is useful to stakeholders?**	⬭	⬭	⬭
**FEASIBLE: Can the evidence gap be filled with a reasonable budget and amount of time?**	⬭	⬭	⬭
**ANSWERABLE: Is the evidence gap well-defined and is the product or endpoint well-framed?3**	⬭	⬭	⬭

99. SC-ANC 6. What are effective ways to improve community knowledge about antenatal self-care?

Mark only one oval per row.

	Yes	No	Don't know
**IMPACTFUL: Would filling this evidence gap provide knowledge that is useful to stakeholders?**	⬭	⬭	⬭
**FEASIBLE: Can the evidence gap be filled with a reasonable budget and amount of time?**	⬭	⬭	⬭
**ANSWERABLE: Is the evidence gap well-defined and is the product or endpoint well-framed?3**	⬭	⬭	⬭

100. SC-ANC 7. Which self-care behaviors during pregnancy are most critical to promote in order to optimize maternal and newborn health?

Mark only one oval per row.

	Yes	No	Don't know
**IMPACTFUL: Would filling this evidence gap provide knowledge that is useful to stakeholders?**	◯	◯	◯
**FEASIBLE: Can the evidence gap be filled with a reasonable budget and amount of time?**	◯	◯	◯
**ANSWERABLE: Is the evidence gap well-defined and is the product or endpoint well-framed?3**	◯	◯	◯

101. SC-ANC 8. How effective is maternal education for self-care?

Mark only one oval per row.

	Yes	No	Don't know
**IMPACTFUL: Would filling this evidence gap provide knowledge that is useful to stakeholders?**	◯	◯	◯
**FEASIBLE: Can the evidence gap be filled with a reasonable budget and amount of time?**	◯	◯	◯
**ANSWERABLE: Is the evidence gap well-defined and is the product or endpoint well-framed?3**	◯	◯	◯

102. SC-ANC 9. How can women be trained to identify danger signs in pregnancy?

Mark only one oval per row.

	Yes	No	Don't know
**IMPACTFUL: Would filling this evidence gap provide knowledge that is useful to stakeholders?**	⬭	⬭	⬭
**FEASIBLE: Can the evidence gap be filled with a reasonable budget and amount of time?**	⬭	⬭	⬭
**ANSWERABLE: Is the evidence gap well-defined and is the product or endpoint well-framed?3**	⬭	⬭	⬭

103. SC-ANC 10. How might pregnant people be provided the self-care technologies, devices, and tools that facilitate self-monitoring during pregnancy?

Mark only one oval per row.

	Yes	No	Don't know
**IMPACTFUL: Would filling this evidence gap provide knowledge that is useful to stakeholders?**	⬭	⬭	⬭
**FEASIBLE: Can the evidence gap be filled with a reasonable budget and amount of time?**	⬭	⬭	⬭
**ANSWERABLE: Is the evidence gap well-defined and is the product or endpoint well-framed?3**	⬭	⬭	⬭

104. SC-ANC 11. How do we standardize automatic blood pressure checking machines for blood pressure checks at home?

Mark only one oval per row.

	Yes	No	Don't know
**IMPACTFUL: Would filling this evidence gap provide knowledge that is useful to stakeholders?**	⬭	⬭	⬭
**FEASIBLE: Can the evidence gap be filled with a reasonable budget and amount of time?**	⬭	⬭	⬭
**ANSWERABLE: Is the evidence gap well-defined and is the product or endpoint well-framed?3**	⬭	⬭	⬭

105. SC-ANC 12. What is the extent of clients' self-care knowledge and skills after completion of pregnancy?

Mark only one oval per row.

	Yes	No	Don't know
**IMPACTFUL: Would filling this evidence gap provide knowledge that is useful to stakeholders?**	⬭	⬭	⬭
**FEASIBLE: Can the evidence gap be filled with a reasonable budget and amount of time?**	⬭	⬭	⬭
**ANSWERABLE: Is the evidence gap well-defined and is the product or endpoint well-framed?3**	⬭	⬭	⬭

106. SC-ANC 13. How can women with high risk pregnancy do fetal kick counts accurately at home?

Mark only one oval per row.

	Yes	No	Don't know
**IMPACTFUL: Would filling this evidence gap provide knowledge that is useful to stakeholders?**			
**FEASIBLE: Can the evidence gap be filled with a reasonable budget and amount of time?**			
**ANSWERABLE: Is the evidence gap well-defined and is the product or endpoint well-framed?3**			

107. SC-ANC 14. What approaches to antenatal self-care are being effectively used, and how can we learn from them?

Mark only one oval per row.

	Yes	No	Don't know
**IMPACTFUL: Would filling this evidence gap provide knowledge that is useful to stakeholders?**	⬭	⬭	⬭
**FEASIBLE: Can the evidence gap be filled with a reasonable budget and amount of time?**	⬭	⬭	⬭
**ANSWERABLE: Is the evidence gap well-defined and is the product or endpoint well-framed?3**	⬭	⬭	⬭

108. SC-ANC 15. What are the limits of antenatal self-care?

Mark only one oval per row.

	Yes	No	Don't know
**IMPACTFUL: Would filling this evidence gap provide knowledge that is useful to stakeholders?**	◯	◯	◯
**FEASIBLE: Can the evidence gap be filled with a reasonable budget and amount of time?**	◯	◯	◯
**ANSWERABLE: Is the evidence gap well-defined and is the product or endpoint well-framed?3**	◯	◯	◯

109. SC-ANC 16. Is self-care during the antenatal period safe?

Mark only one oval per row.

	Yes	No	Don't know
**IMPACTFUL: Would filling this evidence gap provide knowledge that is useful to stakeholders?**	⬭	⬭	⬭
**FEASIBLE: Can the evidence gap be filled with a reasonable budget and amount of time?**	⬭	⬭	⬭
**ANSWERABLE: Is the evidence gap well-defined and is the product or endpoint well-framed?3**	⬭	⬭	⬭

110. SC-ANC 17. Is antenatal self-care cost-effective?

Mark only one oval per row.

	Yes	No	Don't know
**IMPACTFUL: Would filling this evidence gap provide knowledge that is useful to stakeholders?**	◯	◯	◯
**FEASIBLE: Can the evidence gap be filled with a reasonable budget and amount of time?**	◯	◯	◯
**ANSWERABLE: Is the evidence gap well-defined and is the product or endpoint well-framed?3**	◯	◯	◯

111. This survey includes learning questions for five topic areas. Thank you for assessing learning questions on Self-care in the antenatal period. If you would like to assess ANOTHER topic area, please choose one below. You can also end the survey at this point, if you so choose. *

Mark only one oval.

- ◯ Self-managed medical abortion *Skip to question 2*
- ◯ HIV self-testing *Skip to question 30*
- ◯ DMPA-SC *Skip to question 59*
- ◯ Self-care in the antenatal period *Skip to question 94*
- ◯ SRHR self-care general *Skip to question 112*
- ◯ END SURVEY *Skip to question 149*

SRHR self-care general

112. SRHR-SC 1. How do women define self-care?

Mark only one oval per row.

	Yes	No	Don't know
**IMPACTFUL: Would filling this evidence gap provide knowledge that is useful to stakeholders?**			
**FEASIBLE: Can the evidence gap be filled with a reasonable budget and amount of time?**			
**ANSWERABLE: Is the evidence gap well-defined and is the product or endpoint well-framed?**			

113. SRHR-SC 2. Do stakeholders (policymakers/providers) define self-care in the same way as women?

Mark only one oval per row.

	Yes	No	Don't know
**IMPACTFUL: Would filling this evidence gap provide knowledge that is useful to stakeholders?**	⬭	⬭	⬭
**FEASIBLE: Can the evidence gap be filled with a reasonable budget and amount of time?**	⬭	⬭	⬭
**ANSWERABLE: Is the evidence gap well-defined and is the product or endpoint well-framed?**	⬭	⬭	⬭

114. SRHR-SC 3. What are the differentials between individual self-care and self-care involving a friend or family member?

Mark only one oval per row.

	Yes	No	Don't know
**IMPACTFUL: Would filling this evidence gap provide knowledge that is useful to stakeholders?**			
**FEASIBLE: Can the evidence gap be filled with a reasonable budget and amount of time?**			
**ANSWERABLE: Is the evidence gap well-defined and is the product or endpoint well-framed?**			

115. SRHR-SC 4. What is the potential for including menstrual health in self-care strategies?

Mark only one oval per row.

	Yes	No	Don't know
**IMPACTFUL: Would filling this evidence gap provide knowledge that is useful to stakeholders?**	◯	◯	◯
**FEASIBLE: Can the evidence gap be filled with a reasonable budget and amount of time?**	◯	◯	◯
**ANSWERABLE: Is the evidence gap well-defined and is the product or endpoint well-framed?**	◯	◯	◯

116. SRHR-SC 5. Is there any room for sexual and gender-based violence assessments within SRH self-care platforms or tools?

Mark only one oval per row.

	Yes	No	Don't know
**IMPACTFUL: Would filling this evidence gap provide knowledge that is useful to stakeholders?**			
**FEASIBLE: Can the evidence gap be filled with a reasonable budget and amount of time?**			
**ANSWERABLE: Is the evidence gap well-defined and is the product or endpoint well-framed?**			

117. SRHR-SC 6. Is SRHR self-care more important for a particular gender than the other(s)?

Mark only one oval per row.

	Yes	No	Don't know
**IMPACTFUL: Would filling this evidence gap provide knowledge that is useful to stakeholders?**	⬭	⬭	⬭
**FEASIBLE: Can the evidence gap be filled with a reasonable budget and amount of time?**	⬭	⬭	⬭
**ANSWERABLE: Is the evidence gap well-defined and is the product or endpoint well-framed?**	⬭	⬭	⬭

118. SRHR-SC 7. What is the level of people's knowledge about self-care?

Mark only one oval per row.

	Yes	No	Don't know
**IMPACTFUL: Would filling this evidence gap provide knowledge that is useful to stakeholders?**			
**FEASIBLE: Can the evidence gap be filled with a reasonable budget and amount of time?**			
**ANSWERABLE: Is the evidence gap well-defined and is the product or endpoint well-framed?**			

119. SRHR-SC 8. What are people's attitudes toward self-care?

Mark only one oval per row.

	Yes	No	Don't know
**IMPACTFUL: Would filling this evidence gap provide knowledge that is useful to stakeholders?**	⬭	⬭	⬭
**FEASIBLE: Can the evidence gap be filled with a reasonable budget and amount of time?**	⬭	⬭	⬭
**ANSWERABLE: Is the evidence gap well-defined and is the product or endpoint well-framed?**	⬭	⬭	⬭

120. SRHR-SC 9. How can people access factual information about SRHR issues?

Mark only one oval per row.

	Yes	No	Don't know
**IMPACTFUL: Would filling this evidence gap provide knowledge that is useful to stakeholders?**	⬭	⬭	⬭
**FEASIBLE: Can the evidence gap be filled with a reasonable budget and amount of time?**	⬭	⬭	⬭
**ANSWERABLE: Is the evidence gap well-defined and is the product or endpoint well-framed?**	⬭	⬭	⬭

121. SRHR-SC 10. What is the best learning methodology for online SRHR self-care for adult and teens who want information?

Mark only one oval per row.

	Yes	No	Don't know
**IMPACTFUL: Would filling this evidence gap provide knowledge that is useful to stakeholders?**			
**FEASIBLE: Can the evidence gap be filled with a reasonable budget and amount of time?**			
**ANSWERABLE: Is the evidence gap well-defined and is the product or endpoint well-framed?**			

122. SRHR-SC 11. Why and under what circumstances do people choose self-care for SRHR (vs. provider-care)?

Mark only one oval per row.

	Yes	No	Don't know
**IMPACTFUL: Would filling this evidence gap provide knowledge that is useful to stakeholders?**			
**FEASIBLE: Can the evidence gap be filled with a reasonable budget and amount of time?**			
**ANSWERABLE: Is the evidence gap well-defined and is the product or endpoint well-framed?**			

123. SRHR-SC 12. How can we encourage uptake of SHRH self-care services?

Mark only one oval per row.

	Yes	No	Don't know
**IMPACTFUL: Would filling this evidence gap provide knowledge that is useful to stakeholders?**	⬭	⬭	⬭
**FEASIBLE: Can the evidence gap be filled with a reasonable budget and amount of time?**	⬭	⬭	⬭
**ANSWERABLE: Is the evidence gap well-defined and is the product or endpoint well-framed?**	⬭	⬭	⬭

124. SRHR-SC 13. How self-care be made acceptable to women in highly medicalized settings?

Mark only one oval per row.

	Yes	No	Don't know
**IMPACTFUL: Would filling this evidence gap provide knowledge that is useful to stakeholders?**	◯	◯	◯
**FEASIBLE: Can the evidence gap be filled with a reasonable budget and amount of time?**	◯	◯	◯
**ANSWERABLE: Is the evidence gap well-defined and is the product or endpoint well-framed?**	◯	◯	◯

125. SRHR-SC 14. What are the best approaches on male engagement to promote self-care ?

Mark only one oval per row.

	Yes	No	Don't know
**IMPACTFUL: Would filling this evidence gap provide knowledge that is useful to stakeholders?**			
**FEASIBLE: Can the evidence gap be filled with a reasonable budget and amount of time?**			
**ANSWERABLE: Is the evidence gap well-defined and is the product or endpoint well-framed?**			

126. SRHR-SC 15. What can be done to make the promotion of self-care options more appealing to health care workers?

Mark only one oval per row.

	Yes	No	Don't know
**IMPACTFUL: Would filling this evidence gap provide knowledge that is useful to stakeholders?**	◯	◯	◯
**FEASIBLE: Can the evidence gap be filled with a reasonable budget and amount of time?**	◯	◯	◯
**ANSWERABLE: Is the evidence gap well-defined and is the product or endpoint well-framed?**	◯	◯	◯

127. SRHR-SC 16. What are most effective approaches to promoting self-care with policy makers?

Mark only one oval per row.

	Yes	No	Don't know
**IMPACTFUL: Would filling this evidence gap provide knowledge that is useful to stakeholders?**	◯	◯	◯
**FEASIBLE: Can the evidence gap be filled with a reasonable budget and amount of time?**	◯	◯	◯
**ANSWERABLE: Is the evidence gap well-defined and is the product or endpoint well-framed?**	◯	◯	◯

128. SRHR-SC 17. How might we address resistance to self-care, where can we take liberties without obtaining buy-in?

Mark only one oval per row.

	Yes	No	Don't know
**IMPACTFUL: Would filling this evidence gap provide knowledge that is useful to stakeholders?**			
**FEASIBLE: Can the evidence gap be filled with a reasonable budget and amount of time?**			
**ANSWERABLE: Is the evidence gap well-defined and is the product or endpoint well-framed?**			

129. SRHR-SC 18. What support do consumers need to effectively, safely, and confidently uptake self-care?

Mark only one oval per row.

	Yes	No	Don't know
**IMPACTFUL: Would filling this evidence gap provide knowledge that is useful to stakeholders?**			
**FEASIBLE: Can the evidence gap be filled with a reasonable budget and amount of time?**			
**ANSWERABLE: Is the evidence gap well-defined and is the product or endpoint well-framed?**			

130. SRHR-SC 19. Can digital solutions be used to monitor quality of self-care?

Mark only one oval per row.

	Yes	No	Don't know
**IMPACTFUL: Would filling this evidence gap provide knowledge that is useful to stakeholders?**			
**FEASIBLE: Can the evidence gap be filled with a reasonable budget and amount of time?**			
**ANSWERABLE: Is the evidence gap well-defined and is the product or endpoint well-framed?**			

131. SRHR-SC 20. How do we balance SRHR self-care so that people will know when to seek care at a facility?

Mark only one oval per row.

	Yes	No	Don't know
**IMPACTFUL: Would filling this evidence gap provide knowledge that is useful to stakeholders?**	⬭	⬭	⬭
**FEASIBLE: Can the evidence gap be filled with a reasonable budget and amount of time?**	⬭	⬭	⬭
**ANSWERABLE: Is the evidence gap well-defined and is the product or endpoint well-framed?**	⬭	⬭	⬭

132. SRHR-SC 21. Can digital tools link individuals engaged in self-care to the formal health system when/if they need it?

Mark only one oval per row.

	Yes	No	Don't know
**IMPACTFUL: Would filling this evidence gap provide knowledge that is useful to stakeholders?**			
**FEASIBLE: Can the evidence gap be filled with a reasonable budget and amount of time?**			
**ANSWERABLE: Is the evidence gap well-defined and is the product or endpoint well-framed?**			

133. SRHR-SC 22. Are there cost savings to users of self-care?

Mark only one oval per row.

	Yes	No	Don't know
**IMPACTFUL: Would filling this evidence gap provide knowledge that is useful to stakeholders?**			
**FEASIBLE: Can the evidence gap be filled with a reasonable budget and amount of time?**			
**ANSWERABLE: Is the evidence gap well-defined and is the product or endpoint well-framed?**			

134. SRHR-SC 23. Does self-care result in cost savings to the health care system?

Mark only one oval per row.

	Yes	No	Don't know
**IMPACTFUL: Would filling this evidence gap provide knowledge that is useful to stakeholders?**	⬭	⬭	⬭
**FEASIBLE: Can the evidence gap be filled with a reasonable budget and amount of time?**	⬭	⬭	⬭
**ANSWERABLE: Is the evidence gap well-defined and is the product or endpoint well-framed?**	⬭	⬭	⬭

135. SRHR-SC 24. What is the efficacy of SRHR self-care interventions?

Mark only one oval per row.

	Yes	No	Don't know
**IMPACTFUL: Would filling this evidence gap provide knowledge that is useful to stakeholders?**	◯	◯	◯
**FEASIBLE: Can the evidence gap be filled with a reasonable budget and amount of time?**	◯	◯	◯
**ANSWERABLE: Is the evidence gap well-defined and is the product or endpoint well-framed?**	◯	◯	◯

136. SRHR-SC 25. How might we link advancements in individual self-care interventions with broader systems-enablers of self-care (and vice versa)?

Mark only one oval per row.

	Yes	No	Don't know
**IMPACTFUL: Would filling this evidence gap provide knowledge that is useful to stakeholders?**	⬭	⬭	⬭
**FEASIBLE: Can the evidence gap be filled with a reasonable budget and amount of time?**	⬭	⬭	⬭
**ANSWERABLE: Is the evidence gap well-defined and is the product or endpoint well-framed?**	⬭	⬭	⬭

137. SRHR-SC 26. Are providers willing to be a partner in institutionalizing self-care in SRHR?

Mark only one oval per row.

	Yes	No	Don't know
**IMPACTFUL: Would filling this evidence gap provide knowledge that is useful to stakeholders?**			
**FEASIBLE: Can the evidence gap be filled with a reasonable budget and amount of time?**			
**ANSWERABLE: Is the evidence gap well-defined and is the product or endpoint well-framed?**			

138. SRHR-SC 27. What financially sustainable models can be developed to make self-care products available in procurement systems of countries?

Mark only one oval per row.

	Yes	No	Don't know
**IMPACTFUL: Would filling this evidence gap provide knowledge that is useful to stakeholders?**			
**FEASIBLE: Can the evidence gap be filled with a reasonable budget and amount of time?w 2**			
**ANSWERABLE: Is the evidence gap well-defined and is the product or endpoint well-framed? 4**			

139. SRHR-SC 28. How might we integrate self-care into health systems, addressing concerns and ideas of key decision makers?

Mark only one oval per row.

	Yes	No	Don't know
**IMPACTFUL: Would filling this evidence gap provide knowledge that is useful to stakeholders?**	⬭	⬭	⬭
**FEASIBLE: Can the evidence gap be filled with a reasonable budget and amount of time?w 2**	⬭	⬭	⬭
**ANSWERABLE: Is the evidence gap well-defined and is the product or endpoint well-framed? 4**	⬭	⬭	⬭

140. SRHR-SC 29. What is the feasibility and acceptability of an integrated self-care kit for use in emergency response?

Mark only one oval per row.

	Yes	No	Don't know
**IMPACTFUL: Would filling this evidence gap provide knowledge that is useful to stakeholders?**			
**FEASIBLE: Can the evidence gap be filled with a reasonable budget and amount of time?w 2**			
**ANSWERABLE: Is the evidence gap well-defined and is the product or endpoint well-framed? 4**			

141. SRHR-SC 30. What are the barriers and opportunities for advancing SRHR self-care in humanitarian and fragile settings?

Mark only one oval per row.

	Yes	No	Don't know
**IMPACTFUL: Would filling this evidence gap provide knowledge that is useful to stakeholders?**	⬭	⬭	⬭
**FEASIBLE: Can the evidence gap be filled with a reasonable budget and amount of time?w 2**	⬭	⬭	⬭
**ANSWERABLE: Is the evidence gap well-defined and is the product or endpoint well-framed? 4**	⬭	⬭	⬭

142. SRHR-SC 31. To what extent is self-care contributing to Universal Health Coverage (UHC)?

Mark only one oval per row.

	Yes	No	Don't know
**IMPACTFUL: Would filling this evidence gap provide knowledge that is useful to stakeholders?**	⬭	⬭	⬭
**FEASIBLE: Can the evidence gap be filled with a reasonable budget and amount of time?w 2**	⬭	⬭	⬭
**ANSWERABLE: Is the evidence gap well-defined and is the product or endpoint well-framed? 4**	⬭	⬭	⬭

143. SRHR-SC 32. What is the contribution of self-care interventions to improving health care outcomes?

Mark only one oval per row.

	Yes	No	Don't know
**IMPACTFUL: Would filling this evidence gap provide knowledge that is useful to stakeholders?**	◯	◯	◯
**FEASIBLE: Can the evidence gap be filled with a reasonable budget and amount of time?w 2**	◯	◯	◯
**ANSWERABLE: Is the evidence gap well-defined and is the product or endpoint well-framed? 4**	◯	◯	◯

144. SRHR-SC 33. Is self-care a harm reduction intervention?

Mark only one oval per row.

	Yes	No	Don't know
**IMPACTFUL: Would filling this evidence gap provide knowledge that is useful to stakeholders?**			
**FEASIBLE: Can the evidence gap be filled with a reasonable budget and amount of time?w 2**			
**ANSWERABLE: Is the evidence gap well-defined and is the product or endpoint well-framed? 4**			

145. SRHR-SC 34. What are the key indicators for measuring self-care?

Mark only one oval per row.

	Yes	No	Don't know
**IMPACTFUL: Would filling this evidence gap provide knowledge that is useful to stakeholders?**			
**FEASIBLE: Can the evidence gap be filled with a reasonable budget and amount of time?w 2**			
**ANSWERABLE: Is the evidence gap well-defined and is the product or endpoint well-framed? 4**			

146. SRHR-SC 35. What are the measurement challenges of self-care interventions and how can we address them?

Mark only one oval per row.

	Yes	No	Don't know
**IMPACTFUL: Would filling this evidence gap provide knowledge that is useful to stakeholders?**	⬭	⬭	⬭
**FEASIBLE: Can the evidence gap be filled with a reasonable budget and amount of time?w 2**	⬭	⬭	⬭
**ANSWERABLE: Is the evidence gap well-defined and is the product or endpoint well-framed? 4**	⬭	⬭	⬭

147. SRHR-SC 36. Can self-care reduce stigma in SRHR?

Mark only one oval per row.

	Yes	No	Don't know
**IMPACTFUL: Would filling this evidence gap provide knowledge that is useful to stakeholders?**	⬭	⬭	⬭
**FEASIBLE: Can the evidence gap be filled with a reasonable budget and amount of time?w 2**	⬭	⬭	⬭
**ANSWERABLE: Is the evidence gap well-defined and is the product or endpoint well-framed? 4**	⬭	⬭	⬭

148. This survey includes learning questions for five topic areas. Thank you for assessing learning questions on SRHR self-care general. If you would like to assess ANOTHER topic area, please choose one below. You can also end the survey at this point, if you so choose. *

Mark only one oval.

- ⬭ Self-managed medical abortion *Skip to question 2*
- ⬭ HIV self-testing *Skip to question 30*
- ⬭ DMPA-SC *Skip to question 59*
- ⬭ Self-care in the antenatal period *Skip to question 94*
- ⬭ SRHR self-care general *Skip to question 112*
- ⬭ END SURVEY *Skip to question 149*

About you

149. What is your primary affiliation? *

Mark only one oval.

- Multilateral/UN agency
- Government agency or ministry
- Non-governmental organization (NGO)
- Donor agency or foundation
- Academic institution
- Hospital, health clinic, or other health providing organization
- Manufacturer, pharmaceutical company or laboratory
- Consultant / Independent
- Other: ______________________

150. If your affiliation falls into more than one category, what is the second category?

Mark only one oval.

- Multilateral/UN agency
- Government agency or ministry
- Non-governmental organization (NGO)
- Donor agency or foundation
- Academic institution
- Hospital, health clinic, or other health providing organization
- Manufacturer, pharmaceutical company or laboratory
- Consultant / Independent
- Other: ______________________

151. How many years have you worked in your general field? *

Mark only one oval.

- 1-3
- 4-7
- 8-12
- More than 12

152. Which region(s) does your work primarily focus on? *

Check all that apply.

- Asia
- Africa
- Europe
- Latin America and the Carbbean
- North America
- Global

153. What is your level of expertise in SRHR self-care? *

Mark only one oval.

- High
- Moderate
- Little to none

154. Any additional comments

155. Would you be willing to be contacted if we have follow-up questions? *

Mark only one oval.

- Yes *Skip to question 156*
- No
- Maybe *Skip to question 156*

Contact information (optional)

156. Name

____

157. Email address

____

This content is neither created nor endorsed by Google.

Google Forms
